# Supplementary material for: Chemical control of colloidal self-assembly driven by the electrosolvation force
Source: Nat Commun. 2025 Mar 24;16:2872. doi: 10.1038/s41467-025-57953-w (PMC11933346; doi:10.1038/s41467-025-57953-w)
Supplement: Supplementary file 1 — Supplementary Information [file 41467_2025_57953_MOESM1_ESM.pdf]

Supplementary Information

Chemical control of colloidal self-assembly driven by the electrosolvation force

Sida Wang†[1], Rowan Walker-Gibbons†[1], Bethany Watkins[1], Binghui Lin[1], & Madhavi Krishnan*[1,2]

† these authors contributed equally

[1]Physical and Theoretical Chemistry Laboratory, Department of Chemistry, University of Oxford, South Parks Road, Oxford OX1 3QZ, United Kingdom
[2]The Kavli Institute for Nanoscience Discovery, Sherrington Road, Oxford OX1 3QU, United Kingdom.
*correspondence to madhavi.krishnan@chem.ox.ac.uk

Table of Contents

Suppl. Note 1. Further discussion of the interfacial solvation model of particle interactions 6
Suppl. Note 2. Further considerations on particle interactions in alcohols... 9
Suppl. Note 3. Interactions in EG 11
Suppl. Note 4. Interactions in DMSO 12
Suppl. Note 5. Interactions in electrolytes containing zwitterionic amino acids 13
Suppl. Note 6. Simulation trends for electrolytes containing TMAO and TMG................................ 15
Suppl. Note 7. Further considerations in BD simulations.. 16
Suppl. Note 8. Discussion of the surface properties of silica.. 17
Suppl. Note 9. Additional Experiments 18
Supplementary Figures 20
Supplementary Tables... 36
Supplementary references 47

List of Figures

Supplementary Figure 1. Probability distributions of particle diameter for each particle type studied in experiments. 20

Supplementary Figure 2. Particle charge characterization of SiO_2 and NH_2-SiO_2 in aqueous solution, selected solvents and mixtures. 20

Supplementary Figure 3. Simulation setups for studying interfacial solvation at model silica and amine surfaces.. 21

Supplementary Figure 4. Effect of added salt on cluster formation for NH_2, SiO_2 and COOH particles in 2-propanol (IPA). 22

Supplementary Figure 5. Effect of pH on interparticle interactions for NH_2, SiO_2 and COOH particles in 2-propanol (IPA).. 23

Supplementary Figure 6. Effect of solvent pH on interparticle interactions for NH_2 and SiO_2 particles suspended in dimethyl sulfoxide (DMSO). 24

Supplementary Figure 7. Zeta potential distribution for SiO_2 particles in water at various pH. 24

Supplementary Figure 8. Improving agreement between simulated and measured $g(r)$ distributions through the incorporation of a weak external flow in BD simulations. 25

Supplementary Figure 9. Matching the measured $g(r)$ profile for negatively charged SiO_2 particles in EG with BD simulations. 26

Supplementary Figure 10. Total excess dipole moment density, $\mu\mathrm{av}(0)$, in MD simulations of water/zwitterion solutions of varying zwitterion concentration. 27

Supplementary Figure 11. Raw and digitized images of experiments shown in Fig. 2. 28

Supplementary Figure 12. Raw and digitized images of experiments shown in Fig. 3. 28

Supplementary Figure 13. Raw and digitized images of NH_2, SiO_2 and COOH particle interactions in IPA with a variation in added salt. 29

Supplementary Figure 14. Raw and digitized images of NH_2, SiO_2 and COOH particle interactions in IPA with a variation in solution pH. 29

Supplementary Figure 15. Raw and digitized images of NH_2, SiO_2 and COOH particle interactions in water-IPA mixtures 30

Supplementary Figure 16. Raw and digitized images of NH_2 and SiO_2 particle interactions in DMSO with a variation in solution pH. 30

Supplementary Figure 17. Raw and digitized images of NH_2 and SiO_2 particle interactions in EG with variation in solution pH. 31

Supplementary Figure 18. Raw and digitized images of NH_2, SiO_2 and COOH particle interactions in D_2O. 31

Supplementary Figure 19. Raw and digitized images of SiO_2 particle interactions in aqueous solutions containing varying concentrations of Tween 20. 31

Supplementary Figure 20. Raw and digitized images of SiO_2 particle interactions in glycerol solutions with variation in concentration. 32
Supplementary Figure 21. Raw and digitized images of SiO_2, COOH and NH_2 particle interactions in water with a variation in concentration of added TMG. 32
Supplementary Figure 22. Raw and digitized images of SiO_2 particle interactions in TMG-containing water with adjusted pH and conductivity.. 33
Supplementary Figure 23. Raw and digitized images of SiO_2 particle interactions in water containing various concentrations of added TMAO. 33
Supplementary Figure 24. Raw and digitized images of SiO_2 particle interactions in water containing various concentrations of added amino acids... 35
Supplementary Figure 25. Raw and digitized images of SiO_2 and COOH particle interactions in water containing various concentrations of added tyrosine 35

List of Tables

Supplementary Table 1. Detail on solvents used in the study.......36
Supplementary Table 2. Detail on additives used in the study. 36
Supplementary Table 3. List of experimental conditions for experiments shown in Fig. 2. 37
Supplementary Table 4. List of experimental conditions for experiments shown in Fig. 3. 37
Supplementary Table 5. Salt concentration variation experiments for NH_2, SiO_2 and COOH particles in IPA. 38
Supplementary Table 6. pH variation experiments in IPA for NH_2, SiO_2 and COOH particles.......38
Supplementary Table 7. Water-IPA mixture experiments for NH_2, SiO_2 and COOH particles. 39
Supplementary Table 8. pH variation experiments in DMSO for NH_2 and SiO_2 particles.......39
Supplementary Table 9. pH variation experiments in EG for NH_2 and SiO_2 particles. 40
Supplementary Table 10. List of experimental conditions for NH_2, SiO_2 and COOH particle interactions in D_2O. 40
Supplementary Table 11. SiO_2 and COOH particle interaction experiments with varying TMG concentration in water.......41
Supplementary Table 12. pH and conductivity adjusted experiments in TMG-containing water. 41
Supplementary Table 13. SiO_2 particle interactions in water containing TMAO.......42
Supplementary Table 14. SiO_2 particle interactions in water containing L-serine. 42
Supplementary Table 15. SiO_2 particle interactions in water containing glycine.......42
Supplementary Table 16. SiO_2 and COOH particle interactions in water containing L-tyrosine.......42
Supplementary Table 17. SiO_2 particle interactions in water containing L-leucine. 43
Supplementary Table 18. SiO_2 particle interactions in water containing L-phenylalanine. 43
Supplementary Table 19. SiO_2 particle interactions in water containing L-tryptophan. 43
Supplementary Table 20. SiO_2 particle interactions in water containing L-glutamine.......43
Supplementary Table 21. SiO_2 particle interactions in water containing L-proline. 43
Supplementary Table 22. SiO_2 and COOH particle interactions in water containing L-tyrosine.......44
Supplementary Table 23. SiO_2 particle interactions in water containing Tween 20.......44
Supplementary Table 24. SiO_2 particle interactions in water containing glycerol. 44
Supplementary Table 25. $U(x)$ parameter values used in BD simulations to model experiments shown in Fig. 2.......44
Supplementary Table 26. $U(x)$ parameter values modelling experiments in Fig. 3.......45
Supplementary Table 27. $U(x)$ parameter values modelling the experiments shown in Fig. 4. 45
Supplementary Table 28. $U(x)$ parameter values modelling experiments shown in Fig. 6. 45
Supplementary Table 29. $U(x)$ parameter values modelling experiments shown in Fig. 7. 46
Supplementary Table 30. $U(x)$ parameter values modelling experiments shown in Fig. 8 46

Suppl. Note 1. Further discussion of the interfacial solvation model of particle interactions

Previous studies have described in detail the theoretical basis of a working model of the interfacial solvation-driven interparticle interaction – termed the 'interfacial solvation model'[1-3]. Our interfacial-solvation theoretical framework examines the change in system free energy due to interfacial solvent molecules that occurs in response to a decrease in interparticle separation as two particles approach. The mechanism further relies on the fact that the charge carried by particles depends on the pH in solution and is also a function of interparticle separation via a process known as charge regulation[4]. The coupling of charge regulation with the orientational behaviour of the interfacial solvent gives rise to a free energy contribution, ΔF_{int}, which may be added to the traditional electrostatic free energy of interaction, ΔF_{el}, for two charge-regulating particles. A total free energy composed of a sum of these two contributions was found to successfully describe numerous features of the experiment[1,2]. Thus, in essence, the interfacial solvation model presents an attempt to capture the thermodynamic consequences of the symmetry-broken molecular orientational behaviour at an interface that is manifestly absent from continuum theories of electrostatic interactions in the fluid phase.

Within the interfacial solvation model, the effective interparticle interaction potential may be written as

$$U_{\mathrm{tot}} = \Delta F_{\mathrm{el}} + \Delta F_{\mathrm{int}} \approx A\exp\left(-\kappa_1 x\right) + B\exp\left(-\kappa_2 x\right) \qquad (1)$$

where $A > 0$ always, and $\kappa \approx \kappa_1 > \kappa_2$. When $B < 0$ we may expect a long-range attractive force between particles which vanishes for $B = 0$ and turns repulsive for $B > 0$. According to Supplementary Eq. (1), a stable minimum in the pair potential occurs when the canonically repulsive first term dominates at short range ($x < \kappa^{-1}$), since typically, $\frac{A}{|B|} > 1$. But at long range, $x \gg \kappa^{-1}$, since $\kappa_1 > \kappa_2$, the attractive second term competes with the first term and can dominate the overall interaction.

In the original formulation of our theoretical model, we determined the required interfacial free energy in Supplementary Eq. (1) by calculating a charging free energy integral obtained from MD simulations of pure solvents at an interface[1,2]. The simulations relied on inferring the quantity $\varphi_{\mathrm{int}}(\sigma)$, an 'excess' interfacial electrical potential that arises from the solvent alone, for surfaces of various electrical charge density, σ. We then evaluated the interfacial free energy term by integrating the interfacial free energy density over the surface area of two charge-regulating particles whose interaction was analysed using a mean field model of the electrostatics. We thus obtained the following expression for the coefficient of the interfacial free energy term in Supplementary Eq. (1)[1,2]:

$$B \propto ez\varphi_{\mathrm{int}}\Gamma R^2 \frac{\mathrm{d}\alpha}{\mathrm{d}\psi_{\mathrm{s},\infty}}\psi_{\mathrm{s},\infty} \quad (2)$$

In the above equation, e is the elementary charge, $\psi_{\mathrm{s},\infty}$ is the electrical potential at the surface of an isolated particle due to ions in solution, α represents the ionization probability of the particles' chargeable groups whose number density is Γ, and the groups may carry a charge of $z\,e$ in their ionized states where $z = \pm 1$. Importantly, φ_{int} is an excess electrical potential that develops at a surface immersed in a pure solvent, with reference to the electrical potential in the pure bulk solvent[1,2,5].

At the microscopic level, this solvation-related electrical potential arises from the normal component of the net excess solvent molecular dipole moment at an interface with respect to that expected from a continuum picture of a dielectric medium. The symmetry-broken orientational behaviour of solvent molecules at an interface, which gives rise to a net excess dipole moment relative to the bulk, is caused by the presence of an interface that divides the system into two distinct half-spaces and thus introduces an angular anisotropy in hydrogen bonding or other intermolecular interactions of the interfacial molecules. In fact, it is well known from spectroscopic studies and molecular simulations that solvent molecules such as water have a preferred orientation at an interface, even when the surface has zero surface charge[1,5,6]. For water e.g., this preferred orientation arises from a tendency of interfacial water molecules to directionally hydrogen bond towards the bulk liquid rather than the solid interface. However, there are ambiguities involved in inferring a quantity such as φ_{int} from distance dependent profiles of solvent molecular orientation observed in MD simulations[5-9]. In this work we cast the interfacial free energy term in Supplementary Eq. (1) in terms of a net normal excess interfacial dipole moment density which is a quantity that is directly observed in and can be unambiguously inferred from molecular simulations and also from independent experimental methods. In the discussion of this study, we therefore replace the excess interfacial electrical potential, φ_{int}, in Supplementary Eq. (1) with an excess net normal dipole moment surface-density, μ_{av}, where the two quantities are directly related to each other as shown in MD simulation methods (Eq.3).

At a given surface charge density, σ, subtraction of the net dipole moment density in the bulk solvent from the corresponding value in the interfacial region yields $\mu_{\mathrm{av}}(\sigma)$ which thus represents the solvent-density-averaged excess normal component of the molecular dipole moment per unit surface area in the interfacial region. Since $\mu_{\mathrm{av}}(\sigma)$ is directly proportional to $-\varphi_{\mathrm{int}}(\sigma)$, the overall sign and approximate magnitude of the prefactor B can be given in simpler form by:

$$B \propto z\mu_{\mathrm{av}}(\sigma) \quad (3)$$

Thus, in the present study we seek to qualitatively relate the presence or absence of an experimentally observed interparticle attraction in various solvents to the sign of $B = z\mu_{\mathrm{av}}(\sigma)$ expected based on MD simulations. Similar to discussions on φ_{int} in previous work, we will find that $\mu_{\mathrm{av}}(0)$, i.e., the excess dipole moment density obtained in simulations for small values of charge density ($\sigma \to 0$), is sufficient to qualitatively describe the observations. Importantly, we point out that although within the interfacial solvation model $B < 0$ is a necessary condition for interparticle attraction it is not sufficient to support the appearance of a stable minimum ($w < -2\ \mathrm{k_B}T$) in the pair potential which underpins the formation of stable crystalline clusters in experiment. According to Supplementary Eq. (1), very large values of $\frac{A}{|B|} \gg 1$ or small values of $A \to 0$ and $B \to 0$ that arise in the strongly and very weakly charged limits respectively, would not support the formation of stable clusters in solution[1,2]. Since pH exerts significant control of the magnitude of charge carried by protonatable ionisable groups, we may expect cluster formation in experiments to display a strong dependence on pH as shown previously and discussed in detail later[3].

Previous studies provided calculations of pair-interaction potentials under various experimental conditions including salt concentration, pH and the type of solvent, and compared the theoretical predictions with experimentally measured pair-potentials[1-3]. In this study, we limit the scope of data interpretation to providing qualitative assessments of observed experimental trends in relation to the proposed theoretical model of the underlying interaction. We have systematically compared the appearance of clusters, and in some cases the strength of the interparticle attractions underpinning cluster formation, with qualitative indications from the interfacial solvation model. We do not attempt to provide calculations of pair potentials using the interfacial solvation model for the range of experimental conditions described in this work for several reasons: (1) in organic solvents, dissociation constants of the surface ionizable groups (pK values) are not well known, (2) in water-alcohol mixtures, in addition to uncertain pK values[10,11], dielectric constants of the intervening medium are not known, and screening lengths are hard to estimate, (3) in experiments involving osmolytes, parameters underpinning the osmolyte-surface interaction are not well known. Despite the myriad uncertainties on experimental parameters in the systems considered, clear trends that align with the qualitative indications of the interfacial solvation model as given by Supplementary Eq. (3) do nonetheless emerge.

Suppl. Note 2. Further considerations on particle interactions in alcohols

When NH_2 particles were suspended in various short and long-chain alcohols we observed in general the formation of strong, stable clusters which are characteristic of systematically longer screening lengths expected for apolar media that entail a reduction in background ionic strength compared to aqueous media, and in agreement with indications of the interfacial solvation model[2] (Fig. 2,3). Experiments show that at high pH (implying low surface charge densities for basic amine groups), cluster formation is abolished which implies that the deep minimum in the interparticle potential vanishes (Fig. 3). This may be attributed to a reduced charge density and surface potential ψ_s of the interacting particles (reflected in the low measured zeta potentials), a reduction in magnitude of charge regulation as reflected in $|\frac{d\alpha}{d\psi}|$, or both, bearing in mind that each of these effects can independently contribute to $B \to 0$ as given by Supplementary Eq. (2). In general, we found the pH range that promoted cluster formation lay in the region of around 5 to 7 across all the alcohols tested (Supplementary Table 3,4). The pK of amine groups in a high dielectric medium such as water is expected to lie around 9 but may shift to lower values of 7-9 in ethanol, and possibly even lower in media of lower dielectric constant such as longer chain alcohols[11]. As described in previous work, pH values in the vicinity of the pK are expected to result in a substantial contribution from the interfacial free energy term, fostering interparticle attraction and the formation of stable clusters for positive particles in solution[1,2]. At the other extreme, corresponding to low pH, we have an increase in ionic strength due to the added acid (Supplementary Table 6) which may on its own result in dissolution of clusters. Regardless, the particle charge density is higher at lower pH, due to basic ionisable groups, as reflected in higher measured zeta potentials (Supplementary Table 6). As a result, not only does ΔF_{el} increase and compete more strongly with the interfacial term at all separations, but in fact $\mu_{av}(\sigma)$ itself, which is molecule-orientation dependent, may simultaneously decrease in magnitude and may even change sign as the interfacial molecules reorient to point their O atoms preferentially towards an increasingly positively charged surface. This could result in $\mu_{av}(0) \geq 0$ (Fig. 2e) for large and positive σ, which would further reinforce monotonic interparticle electrostatic repulsion, leading to absence of cluster formation at low pH.

On the other hand, negatively charged SiO_2 particles displayed robust interparticle repulsion in alcohols over a range of acidic and basic pH values and following from Supplementary Eq. (3) would indicate $\mu_{av}(0) \leq 0$, similar to that required to explain the cluster formation for positively charged particles. Molecular simulations of the alcohol-solid interface however show that over a range of silanol group densities $\Gamma = (0.5 < \Gamma < 4.7$ nm$^{-2})$ we may expect $0 < \mu_{av}(0) < +1.2$ D nm^{-2} (Fig. 2f). MD simulations of both IPA and1-hexanol at silica surfaces carrying large silanol group densities thus suggest $\mu_{av}(0) > 0$, whose sign does not agree with that of the results obtained for simulations at an

O-atom wall, and in fact points to the possibility of cluster formation for silica particles in alcohols. Our experimental observations however demonstrate the absence of attraction for silica particles in alcohols, indicating either a low magnitude or a negative sign of μ_{av}. The simulation results however do suggest that this condition would be met for net solvent exposed -OH group densities in the range $\Gamma \lesssim 1$ nm^{-2} which may in fact be a reasonable value as discussed in Suppl. Note 5[12-16,17,18].

Suppl. Note 3. Interactions in EG

The balance between gauche and trans conformers in aqueous solutions of EG has been shown to be affected by the dielectric constant of the surrounding medium[19]. Furthermore, ab-initio MD simulations of EG at the air/liquid interface show that EG molecules orient with their hydrophobic methylene groups pointing into the vapour phase[19]. Thus the air/liquid interface is depleted of trans conformers and enriched in gauche conformers with their C-O bonds oriented with the O atom towards the bulk. If the orientational behaviour of molecular EG at the solid/liquid interface were similar to that at the air/liquid interface, we would expect $\mu_{\mathrm{av}}(0) < 0$, similar to the MD simulation result obtained for an O-atom wall. The interfacial solvation model would then suggest attraction between positively charged particles in liquid EG, similar to the results for alcohols (Fig. 2f). Obtaining the correct molecular dipole moment distribution from simulations of EG at the solid/liquid interface would rely crucially on correctly capturing the interfacial conformational and orientational distribution, which in turn is likely to depend strongly on the nature of bonding interactions at the interface. In fact the dramatic difference in sign and magnitude of $\mu_{\mathrm{av}}(0)$ for EG at either O-atom ($\mu_{\mathrm{av}}(0) = -0.5$ D nm^{-2}) or silica surfaces ($\mu_{\mathrm{av}}(0) = -0.1$ to $+0.15$ D nm^{-2}) likely reflects the crucial importance of asymmetric bonding interactions in determining interfacial structural character (Fig. 2f). H-bonding interactions that happen to result in an overall low average value of excess normal dipole moment density, i.e., $\mu_{\mathrm{av}}(\sigma) \rightarrow 0$ would entail $B \rightarrow 0$ in Supplementary Eq. (1), and therefore the absence of cluster formation for both signs of particle charge.

Suppl. Note 4. Interactions in DMSO

Although there is no significant hydrogen bonding in DMSO, there are strong intermolecular dipole-dipole interactions which can lead to anisotropic molecular orientation at an interface[20]. In general we expect $\mu_{\mathrm{av}}(0) < 0$ for DMSO, but for silanol surfaces with values of $\Gamma > 2$ nm^{-2} MD simulations suggest $\mu_{\mathrm{av}}(0) > 0$, which implies $B < 0$ for negatively charged particles, thereby raising the possibility of an attraction for silica particles in DMSO at high silanol or carboxyl group densities[5]. For $\Gamma < 2$ nm^{-2} however we expect $\mu_{\mathrm{av}}(0) \lesssim 0$ and therefore interparticle repulsion for silica particles. Assuming $\mu_{\mathrm{av}} < 0$ to hold for amine surfaces as well, we expect attraction for positively charged particles in DMSO. Note that whilst MD simulations suggest $\mu_{\mathrm{av}}(0) < 0$ for DMSO at aminated surfaces, they also indicate that μ_{av} rapidly decreases in magnitude with increasing positive surface charge density. In fact, surface charge densities $\sigma > 0.15\ e$ nm^{-2} lead to $\mu_{\mathrm{av}} \gtrsim 0$ which would suggest absence of attraction between positively charged particles. These trends are in fact successful in capturing two key features of the experimental observations: (1) relatively weak clustering, since particles typically carry a non-zero net charge density (i.e., $|w|$ is small compared to clustering interactions in other solvents), and (2) that cluster formation occurs over a relatively narrow range of pH compared to alcohols (Supplementary Figure 6).

Suppl. Note 5. Interactions in electrolytes containing zwitterionic amino acids

Amino acids such as phenylalanine, tyrosine, tryptophan and leucine, all carry hydrophobic side chains that are thought to promote adsorption to a silica interface as reported in experiments and MD simulations[22-25] (Fig. 6g). The surface propensity of the hydrophobic amino acids in particular has also been confirmed experimentally using XPS[26]. At the water-air interface, phenylalanine and leucine orient with the hydrophobic moiety towards air and the hydrophilic amino acid functionality exposed to water which favours hydrogen bonding[27]. At the silica-water interface, we may therefore expect a similar driving force for adsorption of these amino acids arising from ordering of the hydrophobic chains at the particle surface away from the bulk water[22,24]. Hydrogen bonding interactions between zwitterionic amino acids and silica surfaces are also known to promote adsorption[28]. Population of the solid-liquid interface by oriented dipolar molecules would immediately suggest an alteration of the net excess interfacial dipole moment. An altered normal component of the excess dipole moment density would be expected to influence the long-range attraction within the interfacial solvation view, potentially furnishing a qualitative explanation of the experimental observations.

In fact, our simulations suggest that not only does $\mu_{\mathrm{av}}(0)$ decrease from its pure-water value and tend to zero with decreasing polarity of the amino acid side chain, but it may even change sign as the polarity of the amino acid side-chain decreases from glycine to tyrosine (Fig. 6f). We note however that the finite size of the simulation box and the need for adequate simulation statistics implies concentrations in our simulations that are 1-2 orders of magnitude higher than the surface concentrations expected based on simulated PMF minima and the experimentally inferred $c_{1/2}$ values (e.g., for tyrosine c_{s} = 40 mM)[23].

Furthermore the study by Bag *et al*. employed a charged silica interface in order to extract the values of the minima in the potentials of mean force, whereas we use a neutral silica surface to infer $\mu_{\mathrm{av}}(0)$ values[23]. Therefore we make use of the PMF values from the study of ref.[23] in Fig. 6 since these results refer to interactions with more realistic model silica surfaces, and we do not attempt to extract PMF values from our simulations that only focus on uncharged hydroxylated silica surfaces. We therefore invoke $\mu_{\mathrm{av}}(0)$ values from our simulations with the sole purpose of outlining qualitative arguments concerning a possible origin of the amino-acid species dependent impact on the experimental observations.

Along these lines, it is not clear, for instance, if under our experimental conditions, $\mu_{\mathrm{av}}(0)$ may realistically be expected to turn significantly negative for tyrosine adsorption as suggested by MD

simulations (Fig. 6f). Importantly, although the simulated $\mu_{\mathrm{av}} < 0$ value obtained for a tyrosine-containing electrolyte at a silica surface might superficially suggest the possibility of an attractive electrosolvation force for positively charged particles, we must also recognize that amino acid adsorption to surfaces is likely to be highly dependent on the chemistry of the surface. Therefore, it does not follow that the adsorption trends expected for silica surfaces and the concomitant impact on μ_{av} could be expected to hold for amine-silica particles. Indeed, clustering of NH_2 particles with amino acids in solution was not observed in experiment (data not shown). Finally, for all simulations involving osmolytes we use a value of $\Gamma = 4.7$ SiOH groups nm^{-2} in line with ref.[23]. Interestingly our simulations of aqueous media containing glycine and tyrosine in contact with a model silica surface carrying an SiOH density of $\Gamma = 1$ nm^{-2} displayed very similar amino acid surface densities ρ_{aa} for both cases when compared to a silica surface with $\Gamma = 4.7$ SiOH groups per nm^2 (Fig. 6g).

In summary, analysis of $\mu_{\mathrm{av}}(0)$ in our MD simulations demonstrates that adsorption of osmolytes at the solid-liquid interface can dramatically alter the structure of the interfacial electrolyte. This disruption to the interfacial electrolyte – smallest for glycine and strongest for tyrosine – correlates well with a significant modulation of the long-ranged attraction between particles in solution.

Suppl. Note 6. Simulation trends for electrolytes containing TMAO and TMG

As stated in the main text, molecular simulations displayed an increase in the interfacial dipole moment density due to water alone, $\mu_{\mathrm{av,w}}$, with increasing concentration of TMG/TMAO in solution. Therefore, solutions containing TMG and TMAO zwitterions appear to have a stronger ordering effect on interfacial water molecules compared to the other zwitterions considered. This feature is supported by other computational and experimental studies and may provide insight into the persistence of cluster formation at high TMG and TMAO concentrations observed in our experiments[29,30]. Interestingly, we also found a similar effect on $\mu_{\mathrm{av,w}}$ for proline at the silica interface, wherein clustering persists at higher concentrations of proline than that suggested solely by trends in affinity for the silica surface of the amino acids examined. Cluster formation in solutions containing the other amino acids followed a bulk concentration dependence that was captured well by the trend in their interfacial binding affinities (Fig. 6,7).

An orientational and hydrogen bond analysis for TMG and TMAO at the silica interface in MD simulations presents a physical picture for the strong ordering effect these zwitterions have on interfacial water molecules (MD Simulation methods). Rather than preferentially hydrogen bonding with the hydrophilic silica surface, TMAO and TMG molecules both orient so that their methyl groups point towards the surface. Their O atoms hydrogen bond with interfacial water molecules with which they form on average ≈ 3 hydrogen bonds per zwitterion at $c_{\mathrm{b}} \approx 3$ M (Fig. 7f). The presence of TMG and TMAO at an interface also causes interfacial water molecules to point their O atoms more on average towards the interface than in the absence of these zwitterions, unlike for most of the other amino acids. As a result the dipole moments of interfacial water molecules in solutions containing TMAO and TMG zwitterions at a silica interface were consistently more aligned with the surface normal over an interfacial region of around 1 nm, resulting in a significantly more positive value of $\mu_{\mathrm{av,w}}(0)$, when compared with pure water (Fig. 7e).

Suppl. Note 7. Further considerations in BD simulations

Incorporating the effect of weak external flows into BD simulations

In experiments it can be challenging to rule out the presence of weak external flows that arise from non-planarity of the cell or evaporation during the observation timeframe. This can result in the formation of large clusters whose size is not captured in our BD simulations that generally do not incorporate these additional effects. One example concerns experiments in 1-hexanol (Fig. 2) where incorporation of particle displacement due to background flow at BD simulation level greatly improves the agreement between the size of clusters in simulations and therefore between measured and simulated $g(r)$s (Supplementary Figure 8)

Matching simulated and measured for SiO_2 particles in EG

In Fig. 2, although particles of neither sign of charge displayed a long-ranged attraction in EG, we noted evidence for an apparently much longer ranged repulsion for negative particles than for positive particles. BD simulations suggest that such a signature in the measured $g(r)$ could arise from strongly heterogeneous interparticle interactions, or alternatively that the pair-interaction in fact reflects a much longer-ranged screened Coulombic decay, captured in this case by a screening length that is a factor ≈20 larger than the nominal Debye length, $\kappa^{-1} \approx 50$ nm (Supplementary Figure 9).

Suppl. Note 8. Discussion of the surface properties of silica

Well hydrated amorphous silica is expected to have a maximum density of $\Gamma = 4.7$ silanol groups per nm^2 - a value reported in experiments and used in simulations[18]. However, several experimental reports of the number density of SiOH are much lower ($\Gamma \leq 1$ group nm^{-2}), with the reported number of deprotonatable sites for amorphous silica even at extremely alkaline pH values of 12 entailing only about 20% of the total nominal group density of $\Gamma =$ 4-5 nm^{-2} [14-17,21]. This suggests an effective accessible SiOH density of $\Gamma < 1$ nm^{-2} groups in a range of spectroscopic experiments that probe the state of the silica interface in contact with the electrolyte. Furthermore, surface silanol groups are present in a variety of forms, supporting different degrees of H-bonding with the solvent, depending on their immediate neighbours e.g., vicinal silanols and geminal groups that can H-bond with each other. Silanol centres carrying a single OH group are classified as Q3, whereas the assignment Q4 describes siloxane bridges where the O atom is engaged only in siloxane bridges and the Si centre lacks an OH group. Importantly mesoporous organosilica particles, prepared by chemical synthesis and similar to those used in this work, have silanol group densities that are strongly dependent on the temperature of any post synthesis treatment with reported values lying between $\Gamma = 0.9$ and 3 nm^{-2} (ref. [13]). In fact ref. [13] shows that the number density of SiOH groups for mesoporous silica can be much less than the total number density of all silanols which include a large fraction of Q4 sites that do not bear an OH group. SiOH groups therefore constitute about 25 to 50% of the total number of all sites (which includes Q2, Q3 and Q4) over a number of different silica preparations.

In our simulations, for simplicity we consider a model silica surface carrying a mix of Q3 and Q4 groups, where a density of $\Gamma = 4.7$ OH nm^{-2} implies 100% Q3 SiOH groups. Since Q3 groups are not expected to be present at these densities on mesoporous silica surfaces, it is likely that this model-surface configuration overestimates the H-bonding ability of our silica microsphere surface, and therefore the impact of the surface on interfacial electrolyte structure. The resulting $\mu_{\mathrm{av}}(0)$ values may therefore be regarded as providing an "upper limit" on the impact of surface H-bonding on the interfacial solvent. Thus, taking into consideration the presence of the various other surface silanol species with different H-bonding character, as well as accounting for the solvent inaccessibility of interior groups that contribute to the total nominal silanol density value, may, together or independently, point to a smaller density of Q3 silanol groups required in our simulation set-up in order to faithfully capture the true net impact of surface chemistry on interfacial solvent structuring relevant to the experiments. Hence we provide $\mu_{\mathrm{av}}(0)$ values for two limiting cases of SiOH group densities in Fig. 2f, namely $\Gamma = 0.5$ and 4.7 OH nm^{-2}, with the trends in our study rather aligning with the indications provided by the lower of the two values.

Suppl. Note 9. Additional Experiments

Cluster formation of COOH particles in osmolyte/water solutions

We briefly discuss the role played by zwitterions in cluster formation of COOH particles. TMG was found to influence the interaction of COOH particles in a similar fashion to that observed for SiO_2, with attractive particle clusters persisting up to high concentrations of about 0.5 M, albeit an order of magnitude lower than the $c_{1/2}$ value noted for silica particles (Supplementary Figure 21). Furthermore, we found that whilst COOH particles displayed similar trends to silica particles for some amino acids, we also observed significantly different cluster forming behaviour than for silica, e.g., in response to a small perturbation in pH (Supplementary Figure 25). Within our view of the electrosolvation force outlined above, disparities in cluster formation behaviour in response to osmolytes and co-solvents in solution are to be expected for chemically different particles. This would follow from the fact that adsorption energies of small molecules and their interactions at the interface are likely to be profoundly dependent on intermolecular interactions as well as molecule-surface interactions that are in turn specifically dependent on the particle surface chemistry and any response thereof to solution conditions. Whilst contributing to the richness and complexity of the underlying phenomenology, the dependence of cluster formation on particle properties, such as surface chemistry, in negatively charged particles (e.g., COOH or SiO_2) directly implicates the interface in dictating the magnitude of the attractive electrosolvation force. Note that in these experiments, the remaining system properties, i.e., sign of the particle charge, pH, salt concentration, and solvent composition or osmolyte type and concentration, remain identical, and only the particle chemistry varies.

Sign inversion of zeta potential for negatively charged particles in IPA under acidic conditions

As shown in Supplementary Table 6 and Supplementary Figure 5, for experiments on particle interactions suspended in IPA at acidic pH values ≤ 5, both SiO_2 and COOH particles displayed a measured positive zeta potential. The origin of this inversion in sign of zeta potential is not clear and could stem from a number of sources, including possible charge reversal, or protonation of the silanol groups due to an increased pK value of silanol groups in alcohols. Counterion governed charge inversion or reversal is traditionally expected in the strong coupling regime which is favoured by multivalent counter-ions and small dielectric constants - conditions which are not met in these experiments[31,32]. In order to indirectly probe the sign of the charge of the particle relevant for an interaction, we studied particle-wall interactions for COOH and SiO_2 particles suspended in IPA containing 0.01 mM HCl. Particle-surface interactions were examined in an observation cell coated with positively charged PEI

polymer, generally used when imaging positively charged NH_2 particles. Interestingly, both COOH and SiO_2 particles, that registered positive zeta potentials, stuck to the positively coated cell surface, as expected rather for negatively charged particles interacting with a PEI coated surface. In contrast, both COOH and SiO_2 particles in protonated IPA diffused freely above an untreated negatively charged glass surface, displaying behaviour typical for negatively charged particles. Taken together, our "surface interaction based" qualitative inference of particle charge suggests that both types of particles in fact most likely carry a negative sign of charge. This observation echoes similar reports on silica particles coated with lipid bilayers of variable sign of charge and density[33]. Furthermore, the same silica particles suspended in water at lower pH $\approx$ 4 also often displayed a large fraction of particles with positive zeta potentials, although silanol groups are generally not expected to exist in the doubly protonated state ($SiOH_2^+$) at weakly acidic pH (Supplementary Figure 7). Zeta potential measurements therefore likely report on additional electrokinetic or interfacial contributions not necessarily reflecting the electrical charge on the particle exclusively.

Supplementary Figures

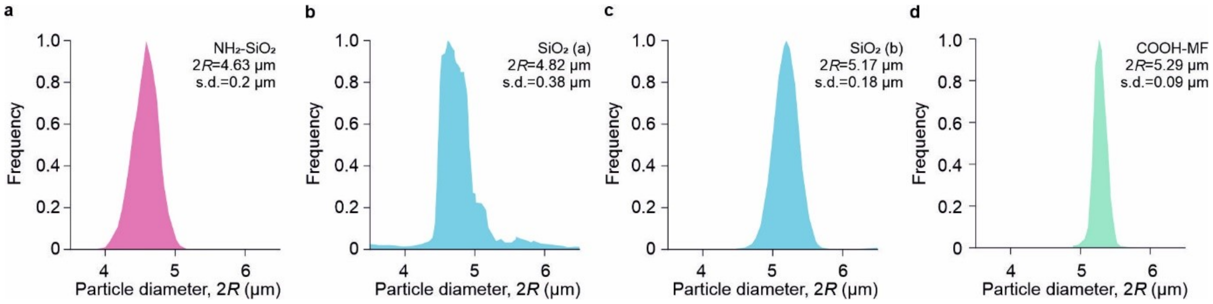

Supplementary Figure 1. Probability distributions of particle diameter for each particle type studied in experiments. a NH_2-SiO_2, **b, c** two types of SiO_2 particles and **d** COOH-MF particles. The distributions were taken from the manufacturer's measurements based on the Coulter principle. The mean and standard deviation (s.d.) of the particle diameter distributions are quoted in the inset.

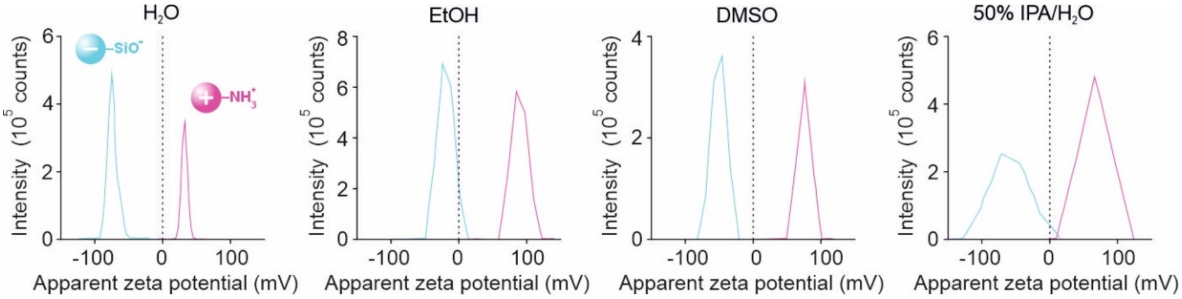

Supplementary Figure 2. Particle charge characterization of SiO_2 and NH_2-SiO_2 in aqueous solution, selected solvents and mixtures. Distribution of zeta potentials measured for particles in H_2O, ethanol, DMSO and 50% IPA/H_2O mixture (left to right). Measurements were performed in the same solution conditions as those used for optical microscopy observation of suspension structures.

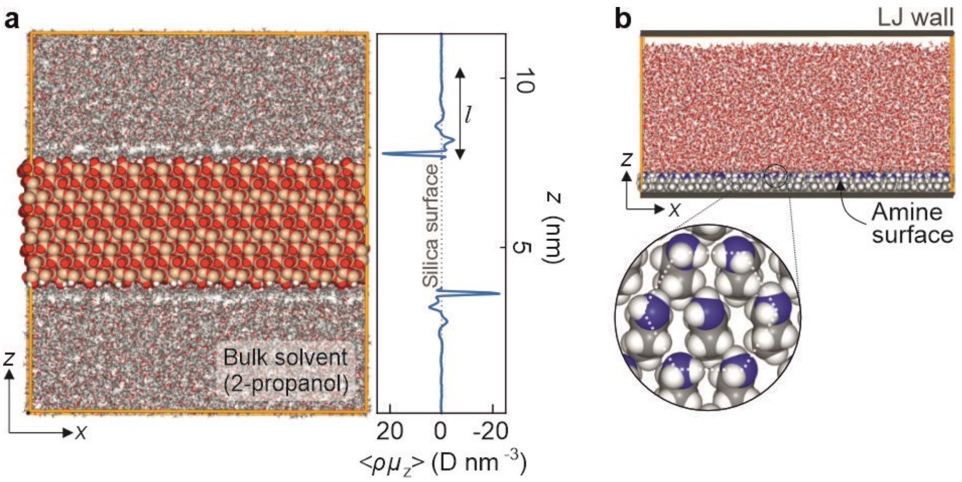

Supplementary Figure 3. Simulation setups for studying interfacial solvation at model silica and amine surfaces. a Simulation cell schematic for all simulations studying the interfacial solvation properties of silica surfaces in this work. An uncharged slab of silica with silanol surface group densities ranging from $\Gamma = 0.5 – 4.7$ OH nm^{-2} generated with the CHARMM-GUI webserver, measuring approximately 10×10 nm^2 in area and 4 nm in width, is solvated on both sides with the fluid phase of interest. Periodic boundaries apply in all three dimensions, as indicated by the yellow box edges. At the interface, solvent molecules display a preferential orientation that results in non-zero average values of the dipole moment density $\langle\rho\mu_\mathrm{z}\rangle$ shown here for 2-propanol (right). The simulation cell is designed to be large enough in the z dimension such that the profiles of the dipole moment density reach a constant value ($\langle\rho\mu_\mathrm{z}\rangle \approx 0$ for an uncharged silica surface in this case) in the bulk liquid, at a distance l which we regard as denoting the approximate thickness of the interfacial region. To calculate $\mu_\mathrm{av}(0)$, the dipole moment density profile is integrated from a location in the bulk liquid to the silica surface as shown in MD simulation methods Eq. (3) and described in detail below. These values are reported in the main text and Fig. 2f. **b** Simulation cell schematic for all simulations studying the interfacial solvation properties of model amine surfaces. Model amine fragments were patterned in a hexagonally close packed arrangement such that the terminal NH_2 group faced the bulk solvent, and were positionally restrained to form the model surface. This surface is placed in contact with the fluid phase of interest, water in the case pictured. At the other end of the simulation cell in the z direction an 'LJ wall' was placed to confine the solvent molecules. Periodic boundaries apply in the xy directions only. Variable NH_2 surface group densities were realised by switching on or off the atomic partial charges of the amine functional groups, whilst retaining their LJ interaction terms.

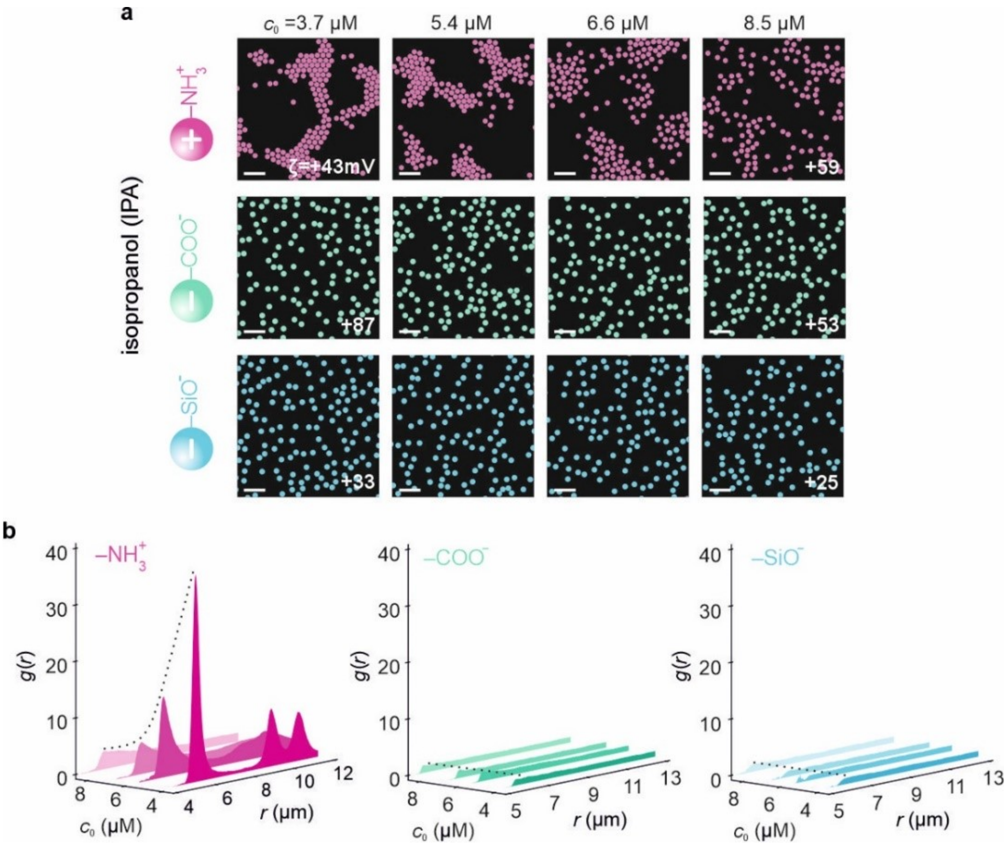

Supplementary Figure 4. Effect of added salt on cluster formation for NH_2, SiO_2 and COOH particles in 2-propanol (IPA). a Digitized experimental image of NH_2, SiO_2 and COOH particle interactions as a function of NaCl concentration in IPA (with 0.01mM HCl). **b** Radial distribution density functions, $g(r)$, as a function of salt concentration for three types of particles. Similar to negatively charged particles in water, interparticle attraction is strongest for positively charged NH_2 particles in IPA containing a lower concentration of salt ($c_0 \approx$ 3-5 μM in this case)[3]. Negatively charged SiO_2 and COOH particles display no evidence of interparticle attraction or cluster formation regardless of the salt concentration. Note that in acidic IPA we measure positive values of zeta potentials for SiO2 and COOH particles which is discussed further below.

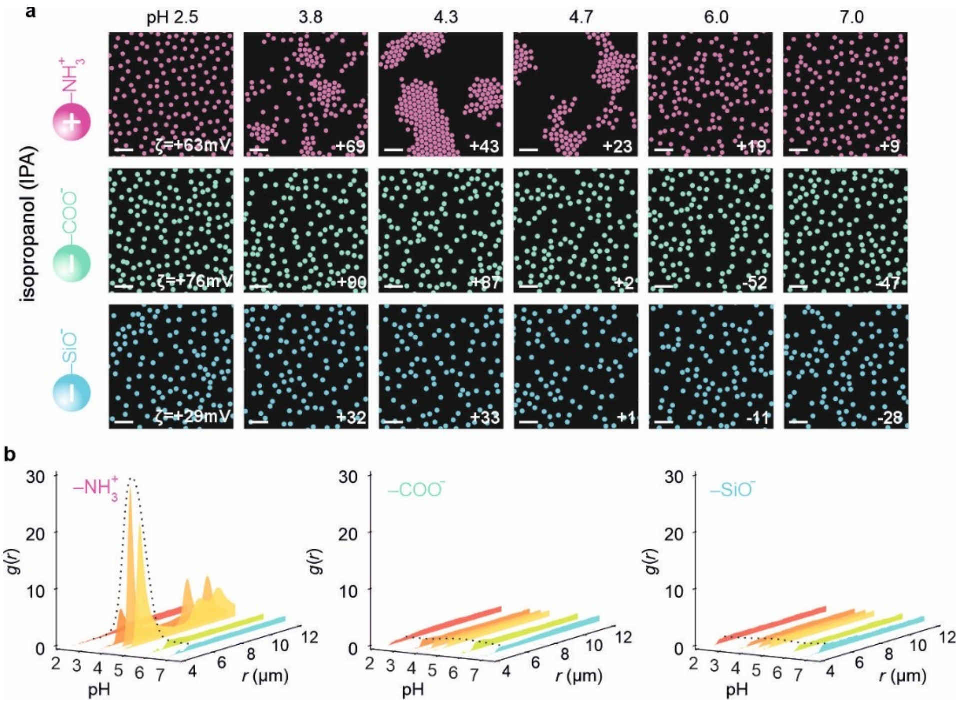

Supplementary Figure 5. Effect of pH on interparticle interactions for NH_2, SiO_2 and COOH particles in 2-propanol (IPA). a Digitized experimental image of NH_2, SiO_2 and COOH particle interactions in IPA as a function of pH. **b** Radial probability density functions, $g(r)$, as a function of pH for three types of particles. We do not maintain constant ionic strength in this set of experiments (Supplementary Table 6). For positively charged NH_2 particles, the strongest interparticle attraction was observed at around pH 4-5. SiO_2 and COOH particles show no evidence of interparticle attraction or cluster formation regardless of pH. Note that for pH<6 we measure positive values of zeta potentials for SiO_2 and COOH particles which is discussed further below.

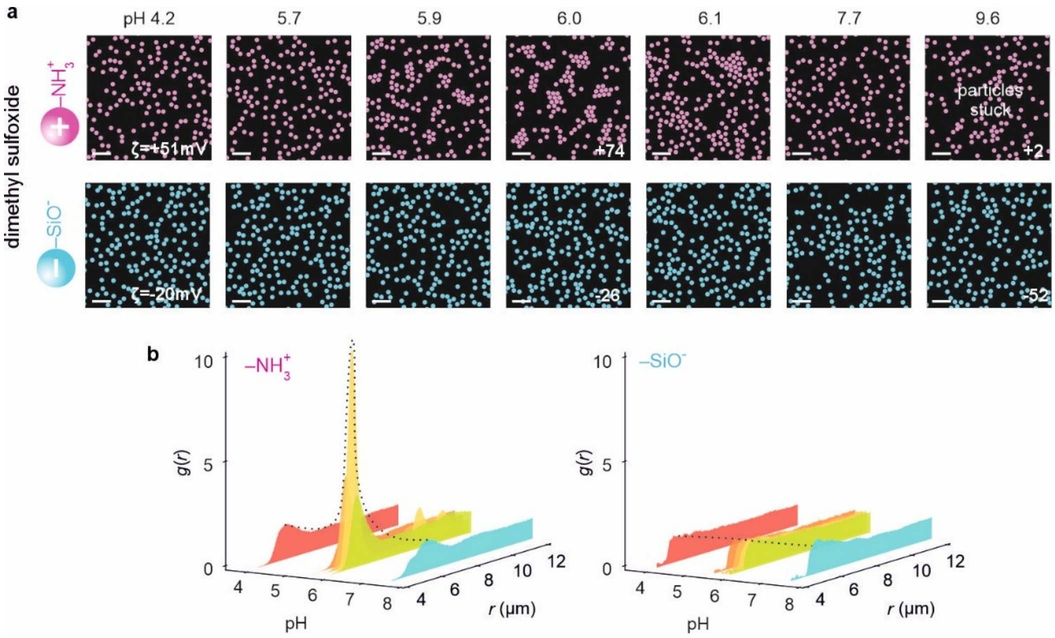

Supplementary Figure 6. Effect of solvent pH on interparticle interactions for NH_2 and SiO_2 particles suspended in dimethyl sulfoxide (DMSO). a Digitized experimental image of NH_2 and SiO_2 particles in DMSO as a function of pH adjusted by the addition of HCl. **b** Radial probability density functions, $g(r)$, as a function of pH for the two types of particles. For positively charged NH_2 particles in DMSO, cluster formation was observed in a narrow range of about 1 pH unit at pH$\approx$ 6. This is in sharp contrast to IPA where cluster formation persisted over a range of ca. 2-3 units of pH (Supplementary Figure 5). No evidence of attraction was found for negatively charged SiO_2 particles in DMSO.

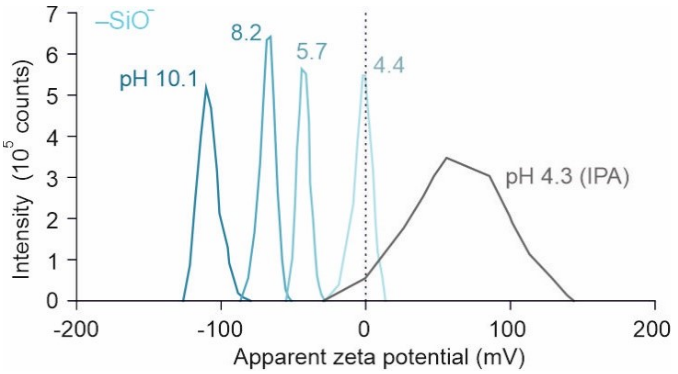

Supplementary Figure 7. Zeta potential distribution for SiO_2 particles in water at various pH. We observe a systematic reduction in zeta potential as pH drops from $\approx$10 to $\approx$4. Four curves plotted from left to right indicate zeta potential distributions with an average of -110 mV, -64 mV, -42 mV and -5 mV in solutions of pH 10.1, 8.2, 5.7 and 4.4, adjusted by the addition of Tris or HCl. For the lowest pH case, a significant fraction of particles appears to be characterized by positive values of zeta potential. The grey curve displays the zeta potential distribution of SiO_2 particles suspended in IPA at pH 4.3, and indicates largely positive values.

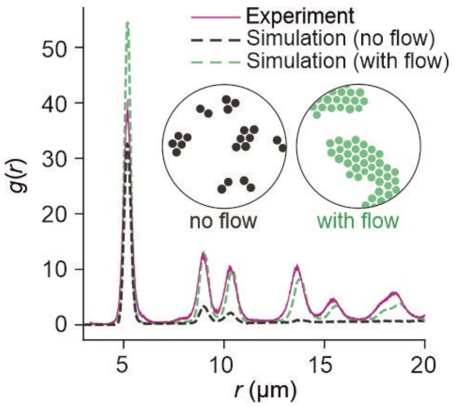

Supplementary Figure 8. Improving agreement between simulated and measured $g(r)$ distributions through the incorporation of a weak external flow in BD simulations. Experimental radial distribution function $g(r)$ for positively charged NH_3^+ particles suspended in 1-hexanol (pink solid line) compared with $g(r)$s outputs from BD simulations that do or do not incorporate the presence of weak external flows (green and grey dashed lines respectively). Snapshots of the simulated suspension structure for the two cases (inset). The pair-interaction potential for both cases is described by Supplementary Eq. (1) with parameters $A = 2482.789\ \mathrm{k_B}T$, $B =$ -2240.343 $\mathrm{k_B}T$, $\kappa_1 = 5.405$ μm^{-1}, $\kappa_2 = 5.135$ μm^{-1}. Particle flow was implemented in the BD simulation by applying a constant force in the x direction of 5 $\mathrm{k_B}T$ μm^{-1} (corresponding to a flow velocity of ≈ 1 μm sec^{-1}) to particles with a total potential energy of interaction greater than -5.5 $\mathrm{k_B}T$. This captures to the first order the influence of weak external flow on cluster formation. Weakly associated or free particles are gradually swept along in the direction of the flow until they encounter a cluster. Here they may engage in strong multivalent nearest-neighbour interactions that anchor them as it were in a growing cluster. In general, this results in the formation of large clusters in simulations, comparable in size to those observed in experiments. The simulated suspension structure then also more closely resembles that seen in experiment (Fig. 2b).

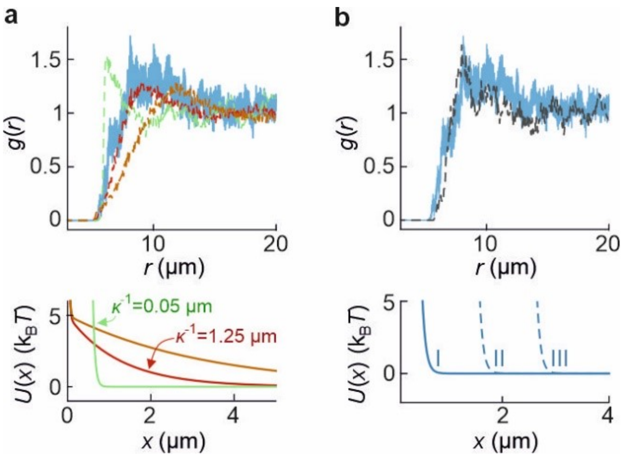

Supplementary Figure 9. Matching the measured $g(r)$ profile for negatively charged SiO_2 particles in EG with BD simulations. **a** Experimental $g(r)$ for SiO_2 particles ($2R$ = 4.82 μm) suspended in EG (blue line). The $g(r)$ profile slowly increases to a maximum value over a distance of a few microns which is markedly different to those measured in other solvents (Fig. 2) and could not be successfully captured using the standard BD approach ($\kappa_1 = \kappa \approx 20$ μm^{-1}, the nominal inverse Debye length), in $U(x)$ equation (green line, Supplementary Table 25 for parameters). However, it is possible to model these observations by assuming a weak and long-ranged repulsive pair-interaction potential $U(x)$ (A =5 k_BT, $\kappa_1 = 0.8$ μm^{-1} and $B = 0$, red line). To prevent particle overlap arising from the weak interparticle repulsion (small A value), a pair potential of the form $U(x) = 30\exp(-50x) + A\exp(-\kappa_1 x)$ is used, where the first term plays the role of a steeply rising repulsive interaction energy mimicking the hard sphere interaction. Another case is presented to highlight the sensitivity of the $g(r)$ to the screening length parameters ($A = 5$ k_BT, κ_1 =0.3 μm^{-1}, $B = 0$, orange line). **b** Matching of the experimental observations by simulating a system with a distribution of A values (Supplementary Eq. (1)). In the simulation, each particle is assigned a particle type I, II or III characterised by a parameter a' = 1×10^4, 1×10^{11} or 1×10^{18} k_BT respectively. Pairwise interactions between particles of type 1 and 2 were characterised by $A = \sqrt{a'_1 a'_2}$ k_BT yielding a pair potential with particle-property-dependent exponential prefactor. Roughly equal numbers of each particle type were present in the simulation. All pair interactions are with a screening of length κ_1 =15 μm^{-1} (inferred from experimental conductivity measurements). Both approaches support the a $g(r)$ profile that slowly increases to a maximum value over the range of a few micrometres in x, and therefore reasonably capture the experimentally observed 'long-range' behaviour. Although there are multiple options to configure BD simulations in a way that enables them to reflect experimental observations, the choice of an appropriate pair potential ultimately depends on knowledge of the underpinning physical mechanisms at play.

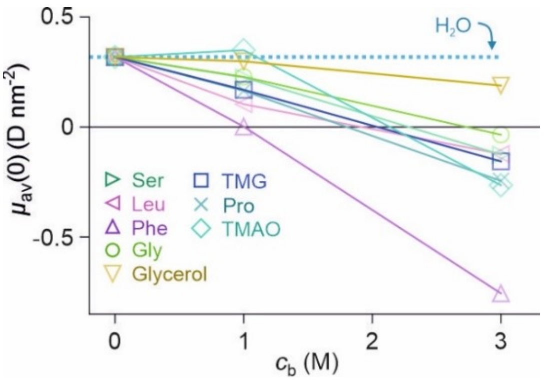

Supplementary Figure 10. Total excess dipole moment density, $\mu_{av}(0)$, in MD simulations of water/zwitterion solutions of varying zwitterion concentration at an uncharged silica surface of silanol group density $\Gamma = 4.7$ OH nm^{-2}. The overall excess dipole moment density includes the contribution of all interfacial molecular species, namely water and osmolyte. Notably, large concentrations of glycerol (up to ca. 1 M) minimally disrupt the value of $\mu_{av}(0)$ such that $\mu_{av}(0) \approx \mu_{av,w}(0)$. Furthermore, the $\mu_{av}(0)$ values characterizing the interfacial electrolyte in solutions containing methylated zwitterions TMAO and TMG show rather similar behaviour to amino acids serine, leucine and glycine. Note however that the $\mu_{av,w}(0)$ values for TMG and TMAO are greatly enhanced compared to their amino acid counterparts (Fig. 7e).

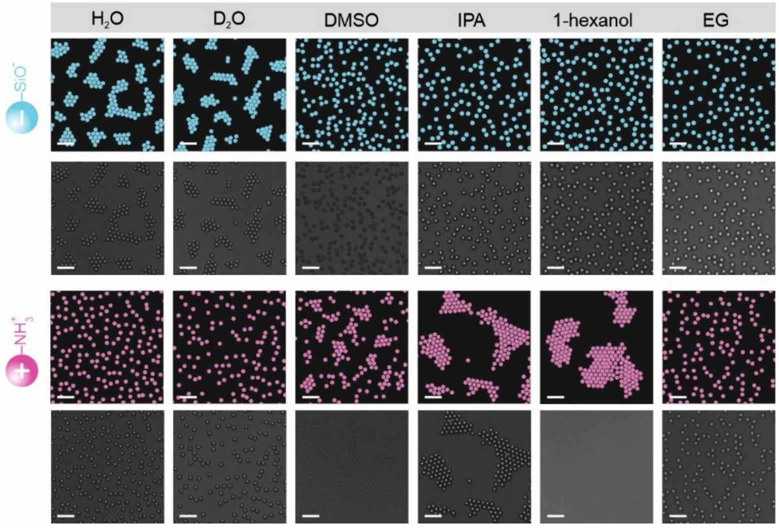

Supplementary Figure 11. Raw and digitized images of experiments shown in Fig. 2.

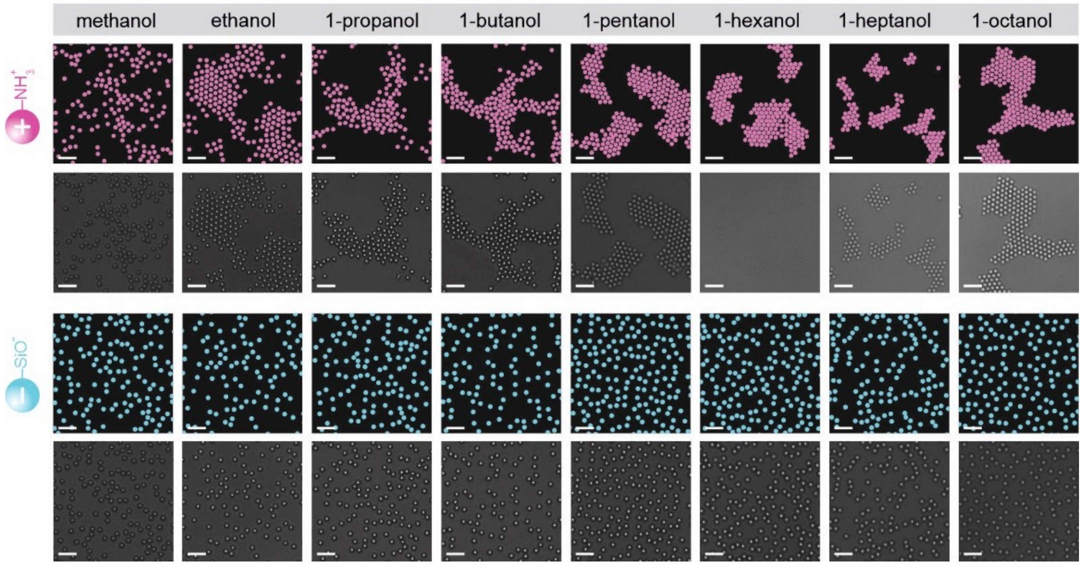

Supplementary Figure 12. Raw and digitized images of experiments shown in Fig. 3.

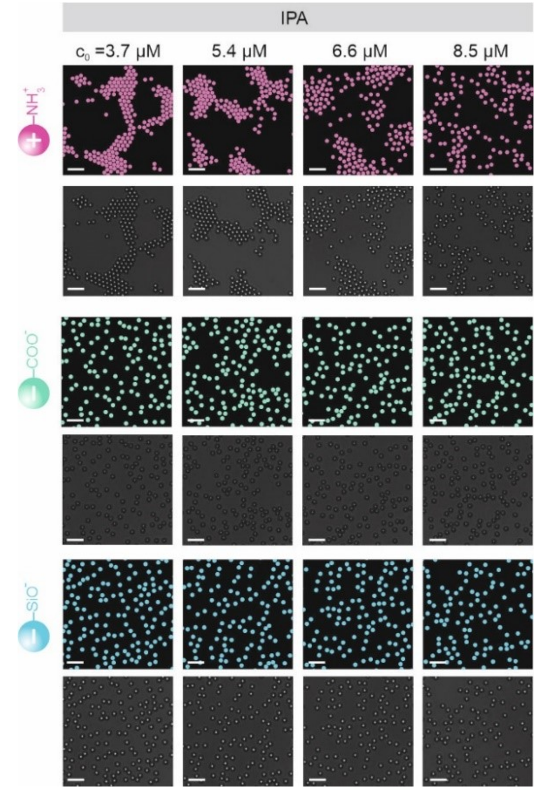

Supplementary Figure 13. Raw and digitized images of NH2, SiO2 and COOH particle interactions in IPA with a variation in added salt.

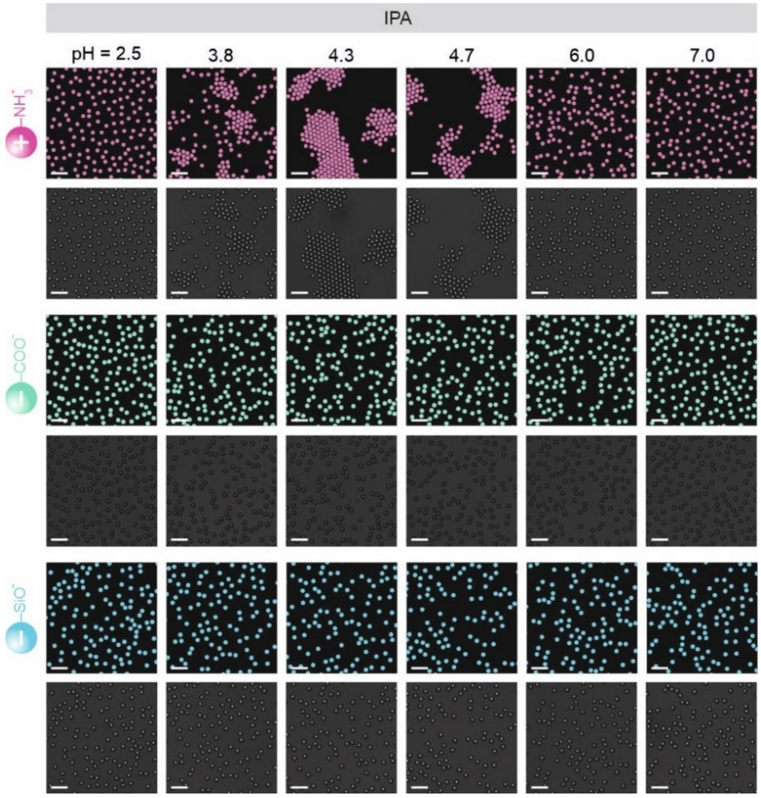

Supplementary Figure 14. Raw and digitized images of NH2, SiO2 and COOH particle interactions in IPA with a variation in solution pH.

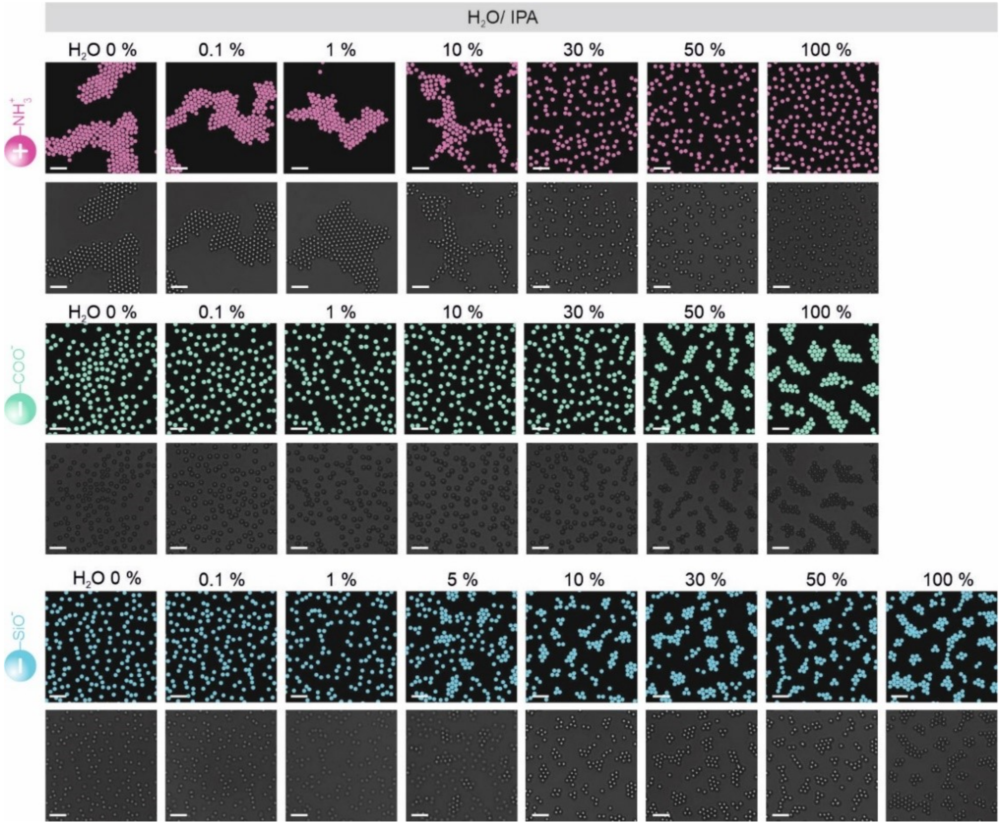

Supplementary Figure 15. Raw and digitized images of NH_2, SiO_2 and COOH particle interactions in water-IPA mixtures (vol.% of H_2O in IPA).

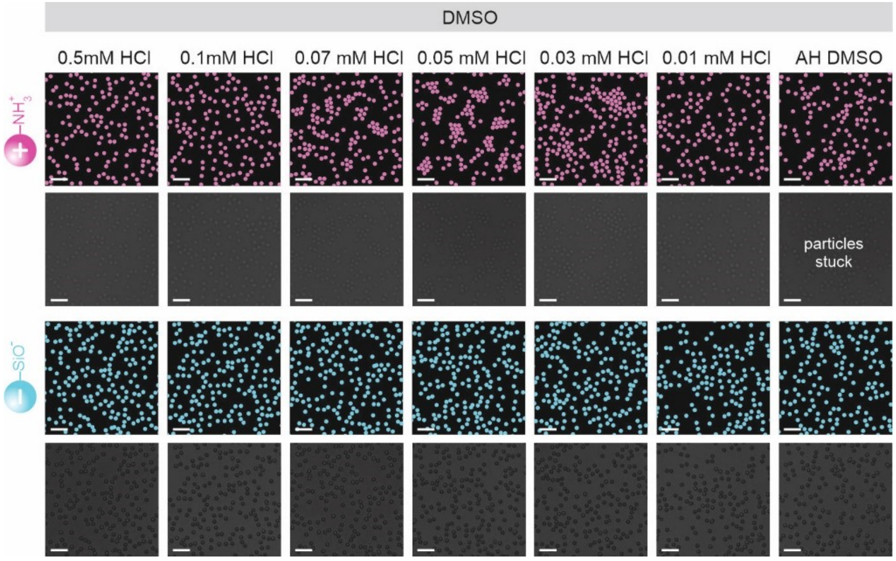

Supplementary Figure 16. Raw and digitized images of NH_2 and SiO_2 particle interactions in DMSO with a variation in solution pH.

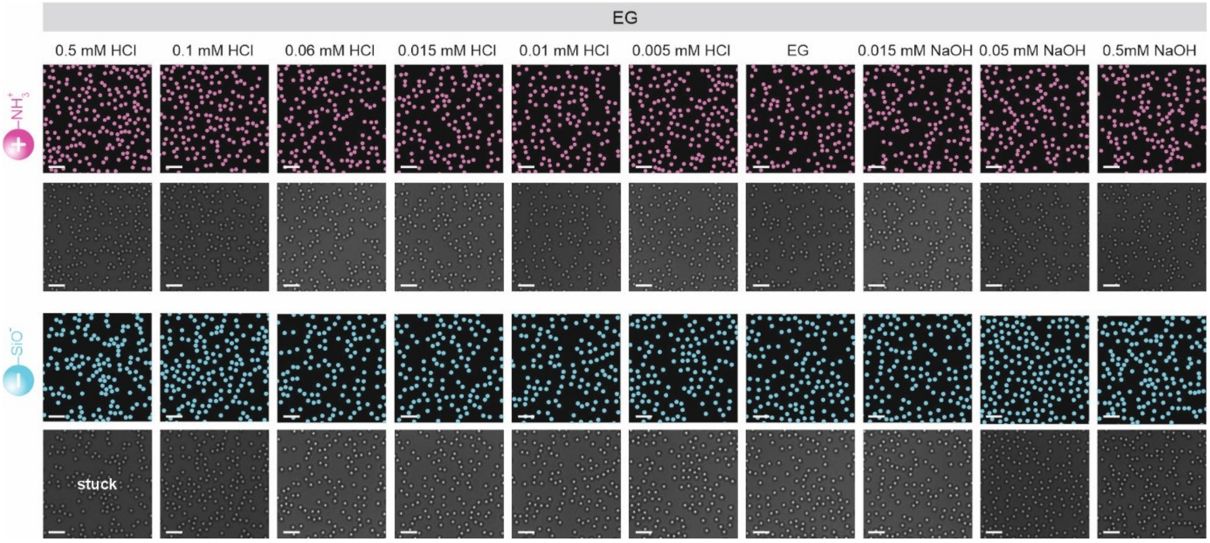

Supplementary Figure 17. Raw and digitized images of NH_2 and SiO_2 particle interactions in EG with variation in solution pH.

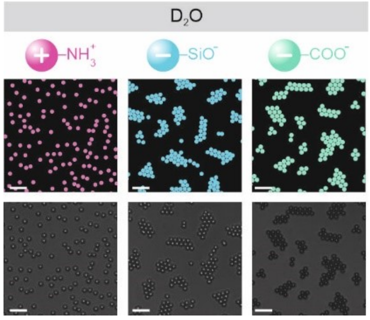

Supplementary Figure 18. Raw and digitized images of NH_2, SiO_2 and COOH particle interactions in D_2O.

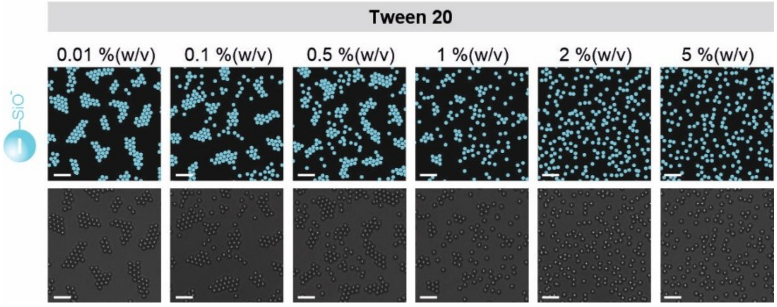

Supplementary Figure 19. Raw and digitized images of SiO_2 particle interactions in aqueous solutions containing varying concentrations of Tween 20 (percentage (w/v) of Tween 20 in water).

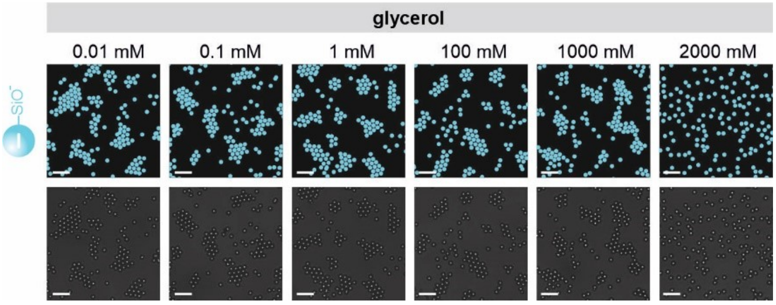

Supplementary Figure 20. Raw and digitized images of SiO_2 particle interactions in glycerol solutions with variation in concentration.

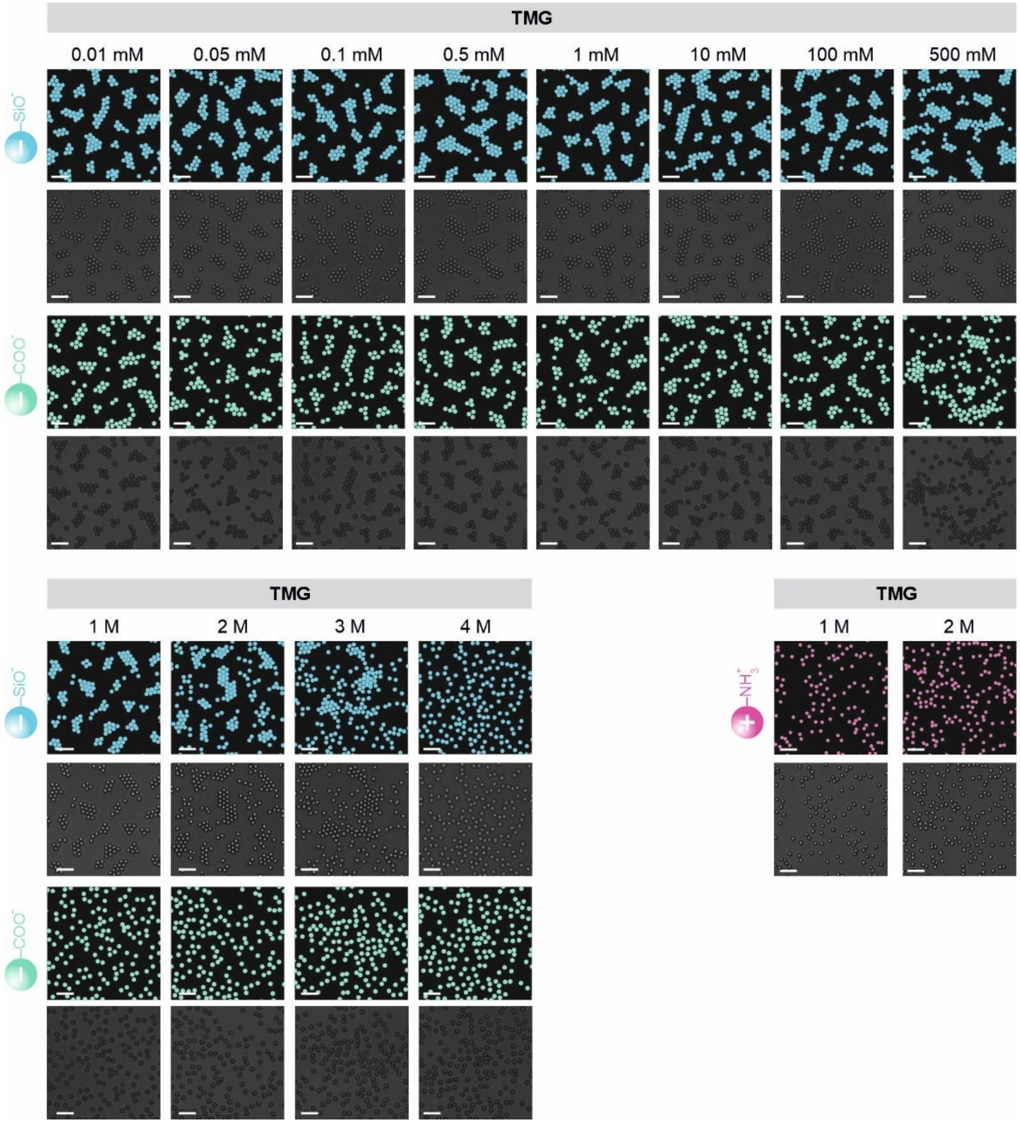

Supplementary Figure 21. Raw and digitized images of SiO_2, COOH and NH_2 particle interactions in water with a variation in concentration of added TMG.

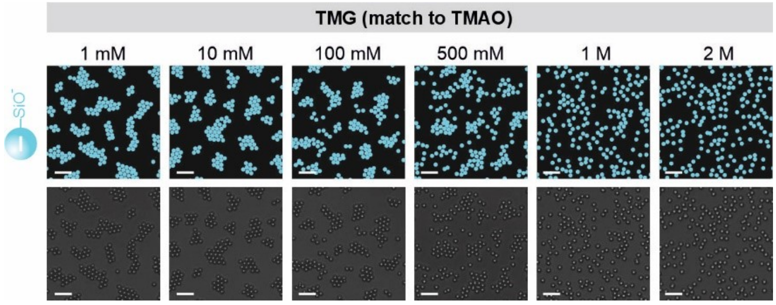

Supplementary Figure 22. Raw and digitized images of SiO_2 particle interactions in TMG-containing water with adjusted pH and conductivity. In each case the pH and conductivity of the TMG-containing electrolyte is adjusted to match that of the corresponding experiment at the same concentration of TMAO (see Supplementary Table 12).

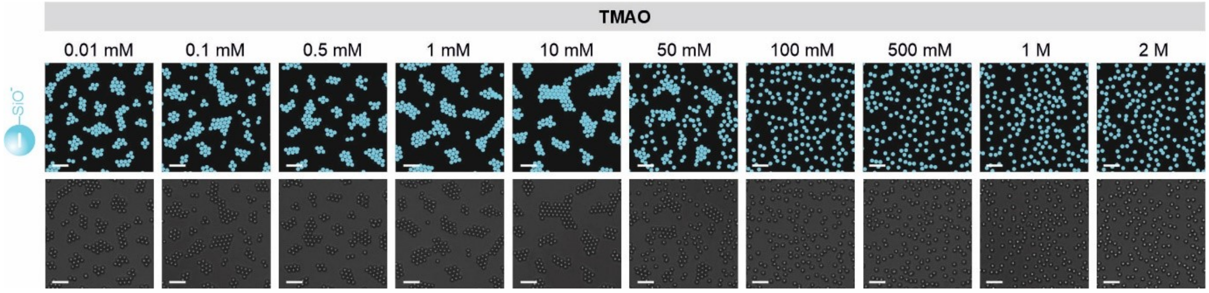

Supplementary Figure 23. Raw and digitized images of SiO_2 particle interactions in water containing various concentrations of added TMAO.

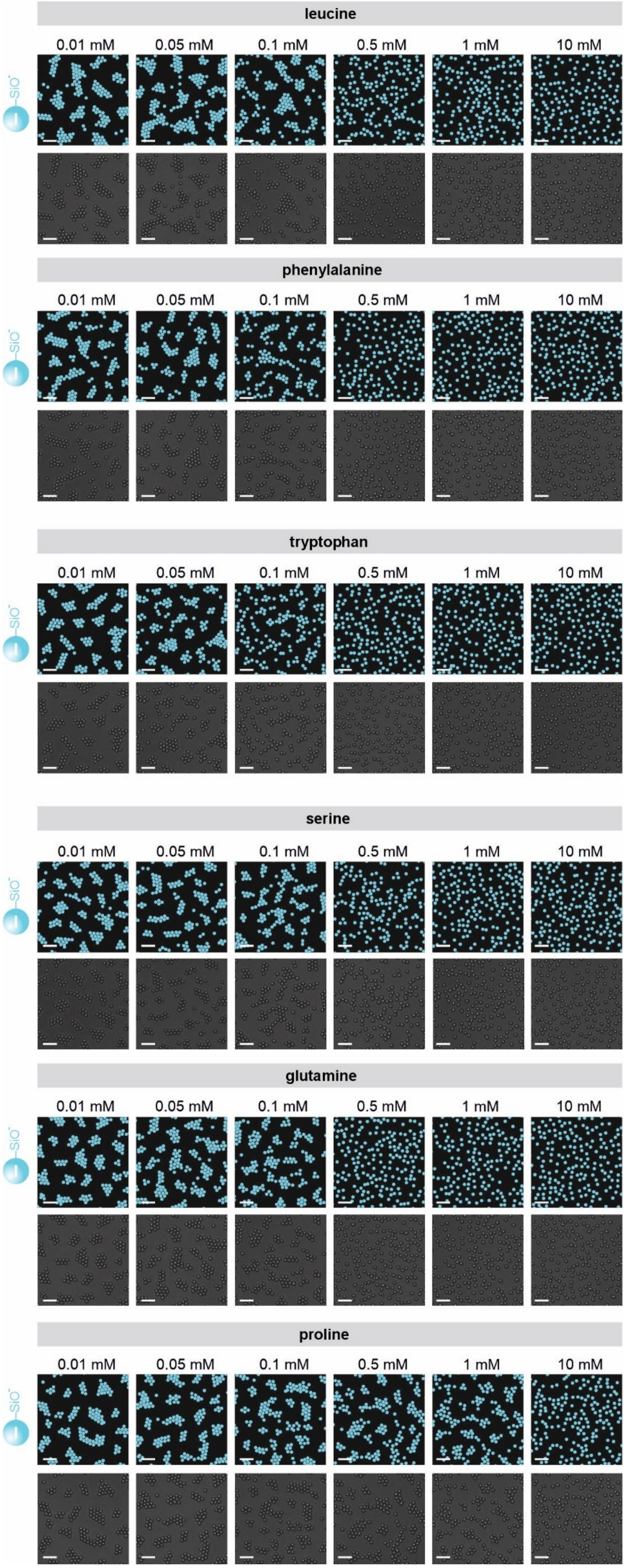
leucine
0.01 mM
0.05 mM
0.1 mM
0.5 mM
1 mM
10 mM
phenylalanine
tryptophan
serine
glutamine
proline
SiO⁻

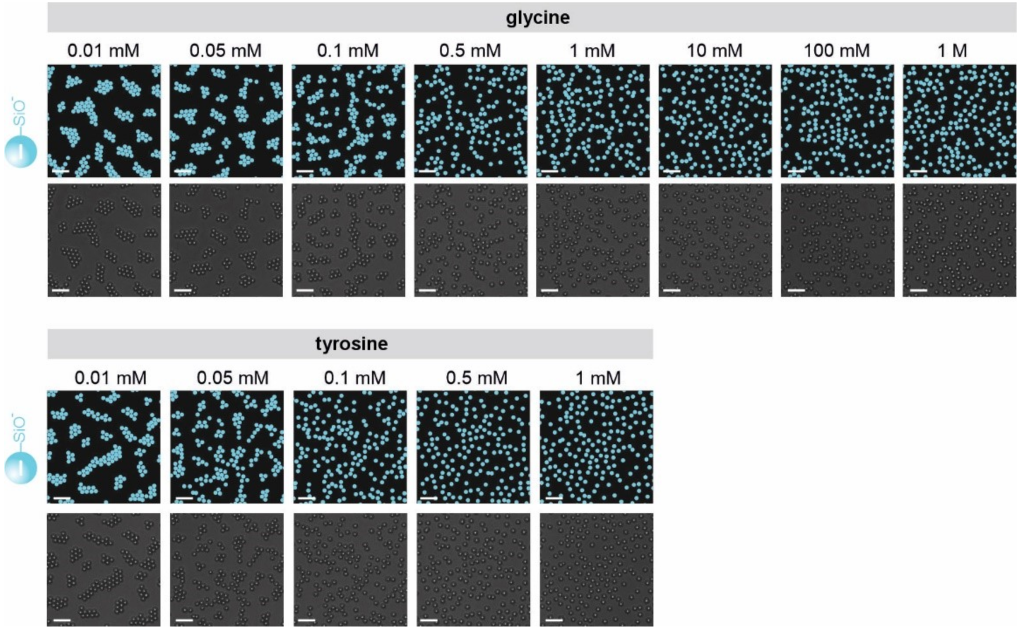

Supplementary Figure 24. Raw and digitized images of SiO_2 particle interactions in water containing various concentrations of added amino acids.

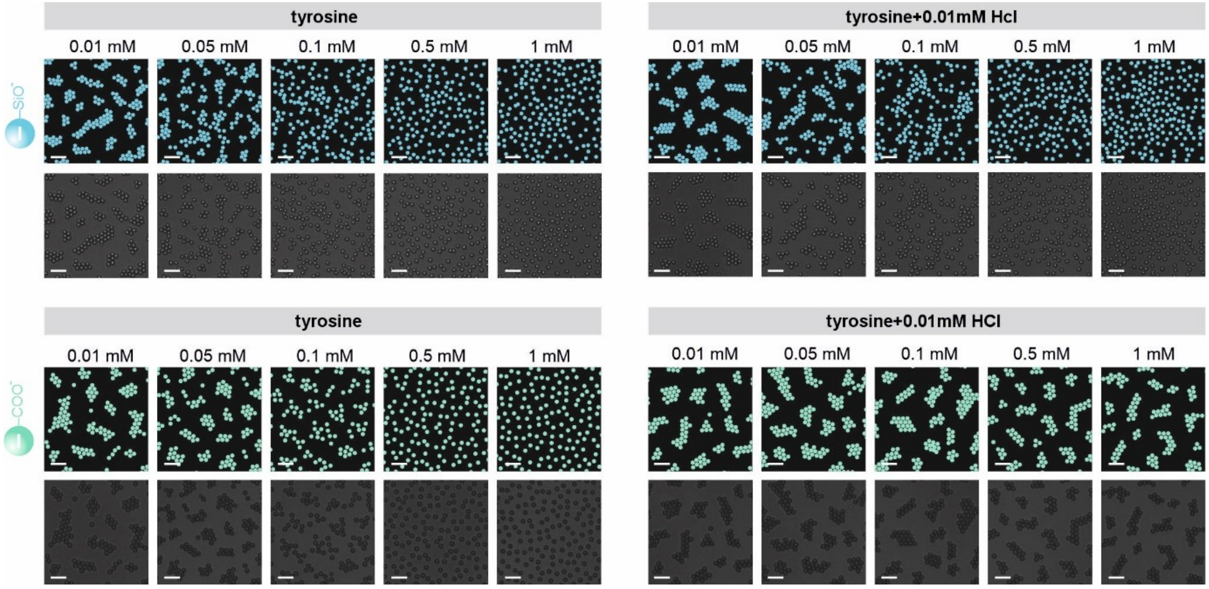

Supplementary Figure 25. Raw and digitized images of SiO_2 and COOH particle interactions in water containing various concentrations of added tyrosine (with and without pH adjustment by the addition of 0.01 mM HCl).

Supplementary Tables

Supplementary Table 1. Detail on solvents used in the study.

Solvent	Product Number	Supplier	Purity
Methanol	34860	Sigma-Aldrich	≥99.9%
Ethanol	32221-M	Sigma-Aldrich	≥99.8%
2-propanol (IPA)	278475	Sigma-Aldrich	anhydrous, 99.5%
1-propanol	279544	Sigma-Aldrich	anhydrous, 99.7%
1-butanol	281549	Sigma-Aldrich	anhydrous, 99.8%
1-pentanol	138975	Sigma-Aldrich	≥99%
1-hexanol	471402	Sigma-Aldrich	anhydrous, ≥99%
1-heptanol	A12793	Thermo Fisher	99%
1-octanol	297887	Sigma-Aldrich	anhydrous, ≥99%
ethylene glycol	324558	Sigma-Aldrich	anhydrous, 99.8%
dimethyl sulfoxide	276855	Sigma-Aldrich	anhydrous, ≥99.9%
deuterium oxide	613444	Sigma-Aldrich	99.9 at.% D

Supplementary Table 2. Detail on additives used in the study.

Additives	Product Number	Supplier	Purity
L-leucine	L8000	Sigma-Aldrich	≥98%
L-phenylalanine	P2126	Sigma-Aldrich	≥98%
L-tyrosine	93829	Sigma-Aldrich	≥99.0%
L-tryptophan	93659	Sigma-Aldrich	≥99.5%
L-serine	S4500	Sigma-Aldrich	≥99%
Glycine	G7126	Sigma-Aldrich	≥99%
L-glutamine	G3126	Sigma-Aldrich	≥99%
L-proline	A10199	Thermo Fisher	99%
Trimethylglycine (TMG)	101003	MP Biomedicals	≥99%
Trimethylamine N-oxide (TMAO)	92277	Sigma-Aldrich	≥99.0%
Tween 20	655206	Sigma-Aldrich	-
Glycerol	G7893	Sigma-Aldrich	≥99.5%
NaCl	010862	Thermo Fisher	99.998%
HCl	087617	Thermo Fisher	99.999%
NaOH	045780	Thermo Fisher	99.99%

Supplementary Table 3. List of experimental conditions for experiments shown in Fig. 2.

Particle	2*R* (µm)	Solvent	pH	Conductivity (µS cm^{-1})	η (cP)	Equivalent c_0 (mM)	ζ (mV)	Added acid/base
NH_2	4.63	H_2O	5.8	1.00	1.0	0.007	30.2±1.7	-
SiO_2	4.82	H_2O	5.8	1.00	1.0	0.007	-70.9±5.3	-
NH_2	4.63	D_2O	6.0	4.04	1.0	0.027	40.3±8.6	-
SiO_2	4.82	D_2O	6.0	4.04	1.0	0.027	-59.3±1.3	-
NH_2	4.63	DMSO	6.0	2.40	2.0	0.032	73.8±2.6	0.05 mM HCl
SiO_2	4.82	DMSO	9.6	0.60	2.0	0.008	-51.8±1.3	-
NH_2	4.63	IPA	4.3	0.25	2.4	0.004	42.7±2.7	0.01 mM HCl
SiO_2	5.17	IPA	6.1	0.08	2.4	0.001	-11.4±0.4	-
NH_2	4.63	1-hexanol	6.3	0.09	6.0	0.003	33.8±2.3	0.01 mM HCl
SiO_2	5.17	1-hexanol	7.3	0.06	6.0	0.002	-24.5±0.7	-
NH_2	4.63	EG	7.1	0.30	15.5	0.031	41.8±2.0	0.01 mM HCl
SiO_2	5.17	EG	8.0	0.28	15.5	0.029	-22.0±5.9	-

Supplementary Table 4. List of experimental conditions for experiments shown in Fig. 3.

Particle	2*R* (µm)	Solvent	pH	Cond. (µS cm^{-1})	η (cP)	Equiv. c_0 (mM)	ζ (mV)	Added acid/base
NH_2	4.63	methanol	6.3	0.73	0.6	0.0029	64.8±13.3	0.01 mM HCl
NH_2	4.63	ethanol	4.3	0.70	1.2	0.0056	81.3±7.1	
NH_2	4.63	1-propanol	7.5	0.64	2.1	0.0088	20.4±10.2	
NH_2	4.63	1-butanol	6.8	0.36	3.0	0.0072	30.0±0.3	
NH_2	4.63	1-pentanol	4.9	0.14	4.3	0.0040	75.2±1.0	
NH_2	4.63	1-hexanol	6.3	0.09	6.0	0.0035	33.8±2.3	
NH_2	4.63	1-heptanol	5.5	0.11	7.8	0.0055	85.7±1.9	
NH_2	4.63	1-octanol	6.0	0.03	10.1	0.0019	47.5±2.8	
SiO_2	5.17	methanol	6.4	0.56	0.6	0.0021	-35.9±8.8	-
SiO_2	5.17	ethanol	6.2	0.02	1.1	0.0001	-18.8±1.2	-
SiO_2	5.17	1-propanol	7.8	0.57	2.3	0.0087	-30.4±2.6	-
SiO_2	5.17	1-butanol	7.9	0.31	3.0	0.0063	-32.1±3.1	-
SiO_2	5.17	1-pentanol	7.0	0.04	4.0	0.0010	-41.0±1.7	-
SiO_2	5.17	1-hexanol	7.3	0.06	5.9	0.0023	-24.5±0.7	-
SiO_2	5.17	1-heptanol	5.3	0.05	7.4	0.0027	-15.9±1.7	-
SiO_2	5.17	1-octanol	6.5	0.01	7.4	0.0007	-25.7±0.9	-

Supplementary Table 5. Salt concentration variation experiments for NH_2, SiO_2 and COOH particles in IPA.

Particle	$2R$ (μm)	Solvent	pH	Cond. (μS cm^{-1})	η (cP)	Equiv. c_0 (mM)	ζ (mV)	Added acid/base
NH_2	4.63	IPA	4.3	0.23	2.4	0.0037	42.7±2.7	0.01 mM HCl
NH_2	4.63	IPA	4.3	0.34	2.4	0.0054	64.5±0.7	
NH_2	4.63	IPA	4.3	0.41	2.4	0.0066	63.2±3.7	
NH_2	4.63	IPA	4.3	0.53	2.4	0.0085	58.6±2.1	
COOH	5.29	IPA	4.3	0.23	2.4	0.0037	87.4±3.5	
COOH	5.29	IPA	4.3	0.34	2.4	0.0054	54.5±1.6	
COOH	5.29	IPA	4.3	0.41	2.4	0.0066	57.0±1.7	
COOH	5.29	IPA	4.3	0.53	2.4	0.0085	53.4±0.8	
SiO_2	5.17	IPA	4.3	0.23	2.4	0.0037	32.6±1.6	
SiO_2	5.17	IPA	4.3	0.34	2.4	0.0054	48.5±2.9	
SiO_2	5.17	IPA	4.3	0.41	2.4	0.0066	42.4±1.7	
SiO_2	5.17	IPA	4.3	0.53	2.4	0.0085	25.2±6.5	

Supplementary Table 6. pH variation experiments in IPA for NH_2, SiO_2 and COOH particles.

Particle	$2R$ (μm)	Solvent	pH	Cond. (μS cm^{-1})	η (cP)	Equiv. c_0 (mM)	ζ (mV)	Added acid/base
NH_2	4.63	IPA	2.5	1.54	2.4	0.0246	63±1	0.06 mM HCl
NH_2	4.63	IPA	3.8	0.4	2.4	0.0064	69±2	0.015 mM HCl
NH_2	4.63	IPA	4.3	0.25	2.4	0.0040	43±3	0.01 mM HCl
NH_2	4.63	IPA	4.7	0.11	2.4	0.0018	23±1	0.005 mM HCl
NH_2	4.63	IPA	6.1	0.08	2.4	0.0013	19±1	-
NH_2	4.63	IPA	7	0.18	2.4	0.0029	9±1	0.015 mM NaOH
NH_2*	4.63	IPA	9.3	0.39	2.4	0.0062	-5±2	0.06 mM NaOH
COOH	5.29	IPA	2.5	1.54	2.4	0.0246	76±6	0.06 mM HCl
COOH	5.29	IPA	3.8	0.4	2.4	0.0064	90±4	0.015 mM HCl
COOH	5.29	IPA	4.3	0.25	2.4	0.0040	87±3	0.01 mM HCl
COOH	5.29	IPA	4.7	0.11	2.4	0.0018	2±1	0.005 mM HCl
COOH	5.29	IPA	6.1	0.08	2.4	0.0013	-52±1	-
COOH	5.29	IPA	7	0.18	2.4	0.0029	-47±2	0.015 mM NaOH
COOH	5.29	IPA	9.3	0.39	2.4	0.0062	-43±2	0.06 mM NaOH
SiO_2	5.17	IPA	2.5	1.54	2.4	0.0246	29±15	0.06 mM HCl
SiO_2	5.17	IPA	3.8	0.4	2.4	0.0064	32±5	0.015 mM HCl
SiO_2	5.17	IPA	4.3	0.25	2.4	0.0040	33±2	0.01 mM HCl
SiO_2	5.17	IPA	4.7	0.11	2.4	0.0018	1±7	0.005 mM HCl
SiO_2	5.17	IPA	6.1	0.08	2.4	0.0013	-11±0	-
SiO_2	5.17	IPA	7	0.18	2.4	0.0029	-28±1	0.015 mM NaOH
SiO_2	5.17	IPA	9.3	0.39	2.4	0.0062	-37±1	0.06 mM NaOH

* rows with grey text indicate cases involving particles stuck to the glass surface (data not used)

** note that the listed measured zeta potentials might not reflect the true sign of surface charge of the particle. See Suppl. Note 10 for details

Supplementary Table 7. Water-IPA mixture experiments for NH_2, SiO_2 and COOH particles.

Particle	$2R$ (μm)	Solvent	Water% (v/v)	pH	Cond. (μS cm^{-1})	η (cP)	Equiv. c_0 (mM)	ζ (mV)	Added acid/base
NH_2	4.63	IPA	0	4.3	0.25	2.4	0.0040	43±2.7	0.01 mM HCl IPA
NH_2	4.63/	H_2O/IPA	0.10	5.8	0.21	2.4	0.0034	68±2.4	
NH_2	4.63	H_2O/IPA	1	5.9	0.23	2.4	0.0036	64±1.6	
NH_2	4.63	H_2O/IPA	10	6.2	0.31	2.2	0.0045	30±4.1	
NH_2	4.63	H_2O/IPA	30	6.2	0.48	1.7	0.0054	35±0.9	
NH_2	4.63	H_2O/IPA	50	5.9	0.75	1.2	0.0060	67±1.6	
NH_2	4.63	H_2O	100	5.8	1.00	1.0	0.0067	30±1.7	
SiO_2	4.82	IPA	0	6.1	0.08	2.4	0.0013	-11±0.4	-
SiO_2	4.82	H_2O/IPA	0.10	7.5	0.12	2.4	0.0019	-9±1.6	
SiO_2	4.82	H_2O/IPA	1	7.3	0.14	2.4	0.0022	-24±1.3	
SiO_2	4.82	H_2O/IPA	5	7.5	0.18	2.3	0.0027	-28±1.0	
SiO_2	4.82	H_2O/IPA	10	7.5	0.20	2.2	0.0029	-28±1.0	
SiO_2	4.82	H_2O/IPA	30	7.3	0.21	1.7	0.0024	-41±3.1	
SiO_2	4.82	H_2O/IPA	50	6.0	0.33	1.2	0.0026	-52±5.7	
SiO_2	4.82	H_2O	100	5.8	1.00	1.0	0.0067	-71±5.3	
COOH	5.29	IPA	0	6.1	0.08	2.4	0.0013	-51±1.8	-
COOH	5.29	H_2O/IPA	0.10	7.5	0.12	2.4	0.0019	-54±2.6	
COOH	5.29	H_2O/IPA	1	7.3	0.14	2.4	0.0022	-56±4.7	
COOH	5.29	H_2O/IPA	10	7.5	0.20	2.2	0.0029	-62±3.5	
COOH	5.29	H_2O/IPA	30	7.3	0.21	1.7	0.0024	-56±2.9	
COOH	5.29	H_2O/IPA	50	6.0	0.33	1.2	0.0026	-99±5.5	
COOH	5.29	H_2O	100	5.8	1.00	1.0	0.0067	-66±1.7	

Supplementary Table 8. pH variation experiments in DMSO for NH_2 and SiO_2 particles.

Particle	$2R$ (μm)	Solvent	pH	Cond. (μS cm^{-1})	η (cP)	Equiv. c_0 (mM)	ζ (mV)	Added acid/base
NH_2	4.63	DMSO	4.2	19.6	2.0	0.2613	51±5	0.5 mM HCl
NH_2	4.63	DMSO	5.7	4.3	2.0	0.0573	-	0.1 mM HCl
NH_2	4.63	DMSO	5.9	3.2	2.0	0.0427	-	0.07 mM HCl
NH_2	4.63	DMSO	6.0	2.4	2.0	0.0320	74±3	0.05 mM HCl
NH_2	4.63	DMSO	6.1	1.67	2.0	0.0223	-	0.03 mM HCl
NH_2	4.63	DMSO	7.7	0.86	2.0	0.0115	-	0.01 mM HCl
NH_2	4.63	DMSO	9.6	0.601	2.0	0.0080	2±5	-
SiO_2	4.82	DMSO	4.2	19.6	2.0	0.2613	-20±4	0.5 mM HCl
SiO_2	4.82	DMSO	5.7	4.3	2.0	0.0573	-	0.1 mM HCl
SiO_2	4.82	DMSO	5.9	3.2	2.0	0.0427	-	0.07 mM HCl
SiO_2	4.82	DMSO	6.0	2.4	2.0	0.0320	-26±2	0.05 mM HCl
SiO_2	4.82	DMSO	6.1	1.67	2.0	0.0223	-	0.03 mM HCl
SiO_2	4.82	DMSO	7.7	0.86	2.0	0.0115	-	0.01 mM HCl
SiO_2	4.82	DMSO	9.6	0.601	2.0	0.0080	-52±0	-

* rows with grey text indicate cases involving particles stuck to the surface (data not used)

Supplementary Table 9. pH variation experiments in EG for NH_2 and SiO_2 particles.

Particle	$2R$ (μm)	Solvent	pH	Cond. (μS cm^{-1})	η (cP)	Equiv. c_0 (mM)	ζ (mV)	Added acid/base
NH_2	4.63	EG	1.2	13.4	15.5	1.3847	-	0.5 mM HCl
NH_2	4.63	EG	3.0	1.4	15.5	0.1447	-	0.1 mM HCl
NH_2	4.63	EG	5.8	0.48	15.5	0.0496	67±6	0.06 mM HCl
NH_2	4.63	EG	7.4	0.28	15.5	0.0289	-	0.015 mM HCl
NH_2	4.63	EG	7.1	0.3	15.5	0.0310	42±2	0.01 mM HCl
NH_2	4.63	EG	7.9	0.28	15.5	0.0289	-	0.005 mM HCl
NH_2	4.63	EG	8.0	0.28	15.5	0.0289	36±3	-
NH_2	4.63	EG	8.0	0.36	15.5	0.0372	-	0.015 mM NaOH
NH_2	4.63	EG	8.3	0.77	15.5	0.0796	27±2	0.05 mM NaOH
NH_2	4.63	EG	9.5	6	15.5	0.6200	-	0.5 mM NaOH
SiO_2	5.17	EG	1.2	13.4	15.5	1.3847	-	0.5 mM HCl
SiO_2	5.17	EG	3.0	1.4	15.5	0.1447	-	0.1 mM HCl
SiO_2	5.17	EG	5.8	0.48	15.5	0.0496	-20±4	0.06 mM HCl
SiO_2	5.17	EG	7.4	0.28	15.5	0.0289	-	0.015 mM HCl
SiO_2	5.17	EG	7.1	0.3	15.5	0.0310	-16±2	0.01 mM HCl
SiO_2	5.17	EG	7.9	0.28	15.5	0.0289	-	0.005 mM HCl
SiO_2	5.17	EG	8.0	0.28	15.5	0.0289	-22±6	-
SiO_2	5.17	EG	8.0	0.36	15.5	0.0372	-	0.015 mM NaOH
SiO_2	5.17	EG	8.3	0.77	15.5	0.0796	-19±4	0.05 mM NaOH
SiO_2	5.17	EG	9.5	6	15.5	0.6200	-	0.5 mM NaOH

* rows with grey text indicate cases involving particles stuck to the surface (data not used).

Supplementary Table 10. List of experimental conditions for NH_2, SiO_2 and COOH particle interactions in D_2O.

Particle	$2R$ (μm)	Solvent	pH	Cond. (μS cm^{-1})	η (cP)	Equiv. c_0 (mM)	ζ (mV)
NH_2	4.63	D_2O	6	4.04	1.0	0.0269	40.3±8.6
COOH	5.29	D_2O	6	4.04	1.0	0.0269	-77.1±1.4
SiO_2	4.82	D_2O	6	4.04	1.0	0.0269	-59.3±1.3

Supplementary Table 11. SiO_2 and COOH particle interaction experiments with varying TMG concentration in water.

Particle	$2R$ (μm)	Additive	c_b (mM)	pH	Cond. (μS cm^{-1})	η (cP)	Equiv. c_0 (mM)	ζ (mV)
SiO_2	4.82	TMG	0	5.8	1	1.0	0.0050	-51±2
SiO_2	4.82	TMG	0.01	5.3	2.3	1.0	0.0153	-64±2
SiO_2	4.82	TMG	0.05	5.3	2.3	1.0	0.0153	-
SiO_2	4.82	TMG	0.1	5.4	2.2	1.0	0.0147	-
SiO_2	4.82	TMG	0.5	5.3	2.1	1.0	0.0140	-
SiO_2	4.82	TMG	1	5.3	2.1	1.0	0.0140	-64±1
SiO_2	4.82	TMG	10	5.3	2	1.0	0.0134	-
SiO_2	4.82	TMG	100	5.6	3.89	1.0	0.0265	-
SiO_2	4.82	TMG	500	6.3	13.9	1.1	0.1036	-
SiO_2	4.82	TMG	1000	6.5	23.5	1.2	0.1949	-57±1
SiO_2	4.82	TMG	2000	6.8	36.1	1.5	0.3665	-
SiO_2	4.82	TMG	3000	7.1	37.6	1.8	0.4601	-
SiO_2	4.82	TMG	4000	7.5	32.5	2.2	0.4729	-30±2
COOH	5.29	TMG	0	5.8	1	1.0	0.0050	-66±2
COOH	5.29	TMG	0.01	5.3	2.3	1.0	0.0153	-59±1
COOH	5.29	TMG	0.05	5.3	2.3	1.0	0.0153	-
COOH	5.29	TMG	0.1	5.4	2.2	1.0	0.0147	-
COOH	5.29	TMG	0.5	5.3	2.1	1.0	0.0140	-
COOH	5.29	TMG	1	5.3	2.1	1.0	0.0140	-60±1
COOH	5.29	TMG	10	5.3	2	1.0	0.0134	-
COOH	5.29	TMG	100	5.6	3.89	1.0	0.0265	-
COOH	5.29	TMG	500	6.3	13.9	1.1	0.1036	-
COOH	5.29	TMG	1000	6.3	23	1.2	0.1908	-64±1
COOH	5.29	TMG	2000	6.8	36.1	1.5	0.3665	-
COOH	5.29	TMG	3000	7.1	37.6	1.8	0.4601	-
COOH	5.29	TMG	4000	7.5	32.5	2.2	0.4729	-

Supplementary Table 12. pH and conductivity adjusted experiments in TMG-containing water.

Particle	$2R$ (μm)	Additive	c_b (mM)	pH	Cond. (μS cm^{-1})	η (cP)	Equiv. c_0 (mM)	ζ (mV)
SiO_2	4.82	TMG	1	6.3	1.82	1.0	0.012	-65±1
SiO_2	4.82	TMG	10	7	5.6	1.0	0.037	-
SiO_2	4.82	TMG	100	7.3	35	1.0	0.239	-71±1
SiO_2	4.82	TMG	500	7.5	122	1.1	0.909	-
SiO_2	4.82	TMG	1000	7.5	214	1.2	1.775	-57±2
SiO_2	4.82	TMG	2000	8.2	280	1.5	2.843	-

Supplementary Table 13. SiO_2 particle interactions in water containing TMAO.

Particle	2*R* (µm)	Additive	c_b (mM)	pH	Cond. (µS cm^{-1})	*η* (cP)	Equiv. c_0 (mM)	*ζ* (mV)
SiO_2	4.82	TMAO	0	5.8	1	1.0	0.005	-51±2
SiO_2	4.82	TMAO	0.01	5.6	1	1.0	0.006	-65±1
SiO_2	4.82	TMAO	0.1	5.7	1	1.0	0.007	-
SiO_2	4.82	TMAO	0.5	6.3	1.3	1.0	0.009	-
SiO_2	4.82	TMAO	1	6.3	1.82	1.0	0.012	-63±2
SiO_2	4.82	TMAO	10	7	5.6	1.0	0.037	-
SiO_2	4.82	TMAO	50	7.4	16.5	1.0	0.111	-
SiO_2	4.82	TMAO	100	7.4	34.5	1.0	0.235	-
SiO_2	4.82	TMAO	500	7.6	124	1.1	0.924	-
SiO_2	4.82	TMAO	1000	7.7	215	1.2	1.783	-41±1
SiO_2	4.82	TMAO	2000	8.2	286	1.5	2.903	-

Supplementary Table 14. SiO_2 particle interactions in water containing L-serine.

Particle	2*R* (µm)	Amino acid	c_b (mM)	pH	Cond. (µS cm^{-1})	*η* (cP)	Equiv. c_0 (mM)	*ζ* (mV)
SiO_2	4.82	L-serine	0.01	5.5	1.21	1.0	0.008	-65±0.6
SiO_2	4.82	L-serine	0.05	5.6	1.23	1.0	0.008	-
SiO_2	4.82	L-serine	0.1	5.7	1.20	1.0	0.008	-66±1.1
SiO_2	4.82	L-serine	0.5	5.7	1.18	1.0	0.008	-
SiO_2	4.82	L-serine	1	5.6	1.19	1.0	0.008	-61±0.8
SiO_2	4.82	L-serine	10	5.7	1.24	1.0	0.008	-77±1.6

Supplementary Table 15. SiO_2 particle interactions in water containing glycine.

Particle	2*R* (µm)	Amino acid	c_b (mM)	pH	Cond. (µS/cm)	*η* (cP)	Equiv. c_0 (mM)	*ζ* (mV)
SiO_2	4.82	glycine	0.01	5.7	1.11	1.0	0.007	-73±2.2
SiO_2	4.82	glycine	0.05	5.8	1.14	1.0	0.008	-
SiO_2	4.82	glycine	0.1	5.6	1.12	1.0	0.007	-61±1.3
SiO_2	4.82	glycine	0.5	5.8	1.17	1.0	0.008	-
SiO_2	4.82	glycine	1	5.6	1.17	1.0	0.008	-61±1.7
SiO_2	4.82	glycine	10	5.8	1.64	1.0	0.011	-82±2.4
SiO_2	4.82	glycine	100	5.9	9.25	1.0	0.063	-78±1.3
SiO_2	4.82	glycine	1000	6.3	70.8	1.2	0.587	-80±0.9

Supplementary Table 16. SiO_2 and COOH particle interactions in water containing L-tyrosine.

Particle	2*R* (µm)	Amino acid	c_b (mM)	pH	Cond. (µS cm^{-1})	*η* (cP)	Equiv. c_0 (mM)	*ζ* (mV)
SiO_2	4.82	L-tyrosine	0.01	5.5	1.17	1.0	0.008	-63±1.5
SiO_2	4.82	L-tyrosine	0.05	5.5	1.22	1.0	0.008	-
SiO_2	4.82	L-tyrosine	0.1	5.5	1.23	1.0	0.008	-62±1.8
SiO_2	4.82	L-tyrosine	0.5	5.5	1.38	1.0	0.009	-
SiO_2	4.82	L-tyrosine	1	5.6	1.50	1.0	0.010	-63±0.9
COOH	5.29	L-tyrosine	0.01	5.5	1.17	1.0	0.008	-67±0.4
COOH	5.29	L-tyrosine	0.05	5.5	1.22	1.0	0.008	-
COOH	5.29	L-tyrosine	0.1	5.5	1.23	1.0	0.008	-63±2.8
COOH	5.29	L-tyrosine	0.5	5.5	1.38	1.0	0.009	-
COOH	5.29	L-tyrosine	1	5.6	1.50	1.0	0.010	-62±1.3

Supplementary Table 17. SiO_2 particle interactions in water containing L-leucine.

Particle	$2R$ (μm)	Amino acid	c_b (mM)	pH	Cond. (μS cm^{-1})	η (cP)	Equiv. c_0 (mM)
SiO_2	4.82	L-leucine	0.01	5.6	1.22	1.0	0.008
SiO_2	4.82	L-leucine	0.05	5.6	1.19	1.0	0.008
SiO_2	4.82	L-leucine	0.1	5.6	1.16	1.0	0.008
SiO_2	4.82	L-leucine	0.5	5.7	1.21	1.0	0.008
SiO_2	4.82	L-leucine	1	5.6	1.16	1.0	0.008
SiO_2	4.82	L-leucine	10	5.6	1.05	1.0	0.007

Supplementary Table 18. SiO_2 particle interactions in water containing L-phenylalanine.

Particle	$2R$ (μm)	Amino acid	c_b (mM)	pH	Cond. (μS cm^{-1})	η (cP)	Equiv. c_0 (mM)
SiO_2	4.82	L-phenylalanine	0.01	5.7	1.18	1.0	0.008
SiO_2	4.82	L-phenylalanine	0.05	5.7	1.19	1.0	0.008
SiO_2	4.82	L-phenylalanine	0.1	5.8	1.21	1.0	0.008
SiO_2	4.82	L-phenylalanine	0.5	5.6	1.20	1.0	0.008
SiO_2	4.82	L-phenylalanine	1	5.5	1.18	1.0	0.008
SiO_2	4.82	L-phenylalanine	10	5.5	1.23	1.0	0.008

Supplementary Table 19. SiO_2 particle interactions in water containing L-tryptophan.

Particle	$2R$ (μm)	Amino acid	c_b (mM)	pH	Cond. (μS/cm)	η (cP)	Equiv. c_0 (mM)
SiO_2	4.82	L-tryptophan	0.01	5.7	1.13	1.0	0.008
SiO_2	4.82	L-tryptophan	0.05	5.7	1.07	1.0	0.007
SiO_2	4.82	L-tryptophan	0.1	5.6	1.24	1.0	0.008
SiO_2	4.82	L-tryptophan	0.5	5.7	1.12	1.0	0.007
SiO_2	4.82	L-tryptophan	1	5.6	1.15	1.0	0.008
SiO_2	4.82	L-tryptophan	10	5.6	1.5	1.0	0.010

Supplementary Table 20. SiO_2 particle interactions in water containing L-glutamine.

Particle	$2R$ (μm)	Amino acid	c_b (mM)	pH	Cond. (μS cm^{-1})	η (cP)	Equiv. c_0 (mM)
SiO_2	4.82	L-glutamine	0.01	5.7	1.19	1.0	0.008
SiO_2	4.82	L-glutamine	0.05	5.6	1.27	1.0	0.008
SiO_2	4.82	L-glutamine	0.1	5.7	1.31	1.0	0.009
SiO_2	4.82	L-glutamine	0.5	5.7	1.37	1.0	0.009
SiO_2	4.82	L-glutamine	1	5.8	1.57	1.0	0.010
SiO_2	4.82	L-glutamine	10	5.7	4.91	1.0	0.033

Supplementary Table 21. SiO_2 particle interactions in water containing L-proline.

Particle	$2R$ (μm)	Amino acid	c_b (mM)	pH	Cond. (μS cm^{-1})	η (cP)	Equiv. c_0 (mM)
SiO_2	4.82	L-proline	0.01	5.8	1.13	1.0	0.008
SiO_2	4.82	L-proline	0.05	5.6	1.18	1.0	0.008
SiO_2	4.82	L-proline	0.1	5.8	1.13	1.0	0.008
SiO_2	4.82	L-proline	0.5	5.8	1.20	1.0	0.008
SiO_2	4.82	L-proline	1	5.9	1.30	1.0	0.009
SiO_2	4.82	L-proline	10	5.9	3.58	1.0	0.024

Supplementary Table 22. SiO_2 and COOH particle interactions in water containing L-tyrosine.

Particle	$2R$ (μm)	Amino acid	c_b (mM)	pH	Cond. (μS cm^{-1})	η (cP)	Equiv. c_0 (mM)	Added acid/base
SiO_2	4.82	L-tyrosine	0.01	4.9	5.53	1.0	0.037	0.01 mM HCl
SiO_2	4.82	L-tyrosine	0.05	4.9	5.35	1.0	0.036	
SiO_2	4.82	L-tyrosine	0.1	4.8	5.3	1.0	0.035	
SiO_2	4.82	L-tyrosine	0.5	4.9	5.43	1.0	0.036	
SiO_2	4.82	L-tyrosine	1	4.8	5.93	1.0	0.040	
COOH	5.29	L-tyrosine	0.01	4.9	5.53	1.0	0.037	
COOH	5.29	L-tyrosine	0.05	4.9	5.35	1.0	0.036	
COOH	5.29	L-tyrosine	0.1	4.8	5.3	1.0	0.035	
COOH	5.29	L-tyrosine	0.5	4.9	5.43	1.0	0.036	
COOH	5.29	L-tyrosine	1	4.8	5.93	1.0	0.040	

Supplementary Table 23. SiO_2 particle interactions in water containing Tween 20.

Particle	$2R$ (μm)	Additive	c_b % (w/v)	pH	Cond. (μS cm^{-1})	η (cP)	Equiv. c_0 (mM)	ζ (mV)	adjustment
SiO_2	4.82	Tween 20	0.01	5.3	1.85	1.0	0.012	-54.5±0.8	-
SiO_2	4.82	Tween 20	0.1	5.3	2.12	1.0	0.014	-37.8±1.6	-
SiO_2	4.82	Tween 20	0.5	4.7	4.67	1.0	0.031	-42.6±0.7	-
SiO_2	4.82	Tween 20	1	4.6	6.64	1.0	0.044	-31.4±0.9	-
SiO_2	4.82	Tween 20	2	4.6	10.02	1.0	0.067	-18.0±0.3	-
SiO_2	4.82	Tween 20	5	4.5	21.46	1.0	0.143	-2.1±0.8	-
SiO_2	4.82	Tween 20	0	4.6	20.65	1.0	0.138	-19.8±2	Control*

* Conductivity and p*H* matched to 5% Tween 20 for comparison

Supplementary Table 24. SiO_2 particle interactions in water containing glycerol.

Particle	$2R$ (μm)	Additive	c_b (mM)	pH	Cond. (μS cm^{-1})	η (cP)	Equiv. c_0 (mM)	ζ (mV)
SiO_2	4.82	glycerol	0.01	5.3	1.24	1.0	1.000	-57.2±0.3
SiO_2	4.82	glycerol	0.1	5.3	1.53	1.0	0.010	-63.2±2.3
SiO_2	4.82	glycerol	1	4.7	1.62	1.0	0.011	-55.4±2.3
SiO_2	4.82	glycerol	100	4.6	2.13	1.0	0.014	-31.4±2.3
SiO_2	4.82	glycerol	1000	4.5	6.50	1.1	0.048	-7.37±0.3
SiO_2	4.82	glycerol	2000	5.9	5.90	1.3	0.051	-5.0±0.4

Supplementary Table 25. $U(x)$ parameter values used in BD simulations to model experiments shown in Fig. 2.

Particle	$2R$ (μm)	Solvent	w (k_BT)	A (k_BT)	B (k_BT)	κ_1 (μm^{-1})	κ_2 (μm^{-1})	A_H (k_BT)
SiO_2	4.82	H_2O	-6	3381.47	-3004.5	8.70	8.27	0.6
SiO_2	4.82	D_2O	-7	8656.562	-7395.153	17.09	16.24	0.6
SiO_2	4.82	DMSO	0	100	-	18.61	-	0.6
SiO_2	5.17	IPA	0	25	-	4.65	-	0.6
SiO_2	5.17	1-hexanol	0	300	-	4.41	-	0.6
SiO_2	5.17	EG*	0	1000000	-	19.74	-	0.6
NH_2	4.63	H_2O	0	1000	-	8.7	-	0.6
NH_2	4.63	D_2O	0	10000	-	17.31	-	0.6
NH_2	4.63	DMSO	-4	5.83E7	-3.12E7	18.61	17.68	0.6
NH_2	4.63	IPA	-5	5255.5	-4526.3	6.58	6.25	0.6
NH_2	4.63	1-hexanol	-6	2482.79	-2240.3	5.40	5.14	0.6
NH_2	4.63	EG	0	1000	-	19.74	-	0.6

* EG data not captured with the standard BD approach, see Supplementary Figure 9 for details.

Supplementary Table 26. $U(x)$ **parameter values modelling experiments in Fig. 3**.

Particle	$2R$ (μm)	Solvent	w (k_BT)	A (k_BT)	B (k_BT)	κ_1 (μm^{-1})	κ_2 (μm^{-1})	A_H (k_BT)
NH_2	4.63	methanol	-1	19808.2	-14730.5	8.393	7.973	0.6
NH_2	4.63	1-propanol	-3.5	2.7E6	-1.7E6	20.395	19.375	0.6
NH_2	4.63	1-pentanol	-7	4.8E6	-3.0E6	14.363	13.646	0.6
NH_2	4.63	1-heptanol	-8	1.2E6	-831574	19.431	18.459	0.6
SiO_2	5.17	methanol	0	100	-	8.393	-	0.6
SiO_2	5.17	1-propanol	0	100	-	20.395	-	0.6
SiO_2	5.17	1-pentanol	0	100000	-	14.363	-	0.6
SiO_2	5.17	1-heptanol	0	100	-	19.431	-	0.6

Supplementary Table 27. $U(x)$ **parameter values modelling the experiments shown in Fig. 4**.

Particle	$2R$ (μm)	Water% (v/v)	w (k_BT)	A (k_BT)	B (k_BT)	κ_1 (μm^{-1})	κ_2 (μm^{-1})	A_H (k_BT)
SiO_2	4.82	0	-0.5	878.543	-737.458	6.579	6.245	0.6
SiO_2	4.82	1	-1	1125.828	-966.301	6.241	5.929	0.6
SiO_2	4.82	5	-3.5	1026.552	-942.387	5.504	5.229	0.6
SiO_2	4.82	10	-4	423.572	-409.165	6.978	6.629	0.6
SiO_2	4.82	30	-4.5	577.178	-552.229	8.057	7.655	0.6
SiO_2	4.82	50	-4.5	577.178	-552.229	8.057	7.655	0.6
SiO_2	4.82	100	-6	857.616	-816.112	8.515	8.089	0.6
NH_2	4.63	0	-6	5332.595	-4631.410	6.579	6.250	0.6
NH_2	4.63	10	-3	4376.839	-3708.282	6.978	6.629	0.6
NH_2	4.63	30	0	25	-	7.644	-	0.6
NH_2	4.63	50	0	100	-	12.04	-	0.6
NH_2	4.63	100	0	300	-	8.515	-	0.6
COOH	5.29	0	0	25	-	6.579	-	0.6
COOH	5.29	10	0	25	-	6.95	-	0.6
COOH	5.29	30	-0.5	169.236	-154.252	8	7.6	0.0
COOH	5.29	50	-3.5	564.658	-534.089	6.90	6.555	0.6
COOH	5.29	100	-5	7436.824	-6294.782	8.515	8.089	0.6

Supplementary Table 28. $U(x)$ **parameter values modelling experiments shown in Fig. 6**.

Particle	$2R$ (μm)	Additive	c_b (mM)	w (k_BT)	A (k_BT)	B (k_BT)	κ_1 (μm^{-1})	κ_2 (μm^{-1})	A_H (k_BT)
SiO_2	4.82	L-serine	0.01	-4.5	1277.001	-1174.238	9.304	8.839	0.6
SiO_2	4.82	L-serine	0.05	-4	1308.764	-1194.910	9.304	8.839	0.6
SiO_2	4.82	L-serine	0.1	-4	735.770	-691.389	9.304	8.839	0.6
SiO_2	4.82	L-serine	0.5	-1.5	12058.534	-9380.984	9.304	8.839	0.6
SiO_2	4.82	L-serine	1	0	50	-	9.304	-	0.6
SiO_2	4.82	L-tyrosine	0.01	-4	1386.478	-1262.219	9.304	8.839	0.6
SiO_2	4.82	L-tyrosine	0.05	-3	923.970	-846.146	9.304	8.839	0.6
SiO_2	4.82	L-tyrosine	0.1	-2	1569.120	-1371.320	9.304	8.839	0.6
SiO_2	4.82	L-tyrosine	1	0	100	-	10.402	-	

Supplementary Table 29. $U(x)$ **parameter values modelling experiments shown in Fig. 7**.

Particle	$2R$ (μm)	Additive	c_b (mM)	w (k_BT)	A (k_BT)	B (k_BT)	κ_1 (μm^{-1})	κ_2 (μm^{-1})	A_H (k_BT)
SiO_2	4.82	TMG	0.01	-5.5	684.884	-656.249	7.355	6.988	0.6
SiO_2	4.82	TMG	1	-5.5	1800.91	-1644.18	12.308	11.693	0.6
SiO_2	4.82	TMG	100	-5.5	3732.97	-3286.13	17.093	16.238	0.6
SiO_2	4.82	TMG	1000	-6.5	58193.6	-45028.9	45.935	43.638	0.6
SiO_2	4.82	TMG	2000	-6	453276	-315258	62.931	59.785	0.6
SiO_2	4.82	TMG	3000	-4.5	9.7E9	-4.0E9	67.575	64.196	0.6
SiO_2	4.82	TMG	4000	0	100	0	66.931	63.584	0.6
SiO_2	4.82	TMAO	0.01	-5.5	721.537	-689.569	8.058	7.654	0.6
SiO_2	4.82	TMAO	1	-5.5	1958.63	-1780.68	11.395	10.825	0.6
SiO_2	4.82	TMAO	10	-6	3360.13	-2986.48	20.009	19.009	0.6
SiO_2	4.82	TMAO	50	-4.5	434091	-298247	34.657	32.924	0.6
SiO_2	4.82	TMAO	100	0	100	0	50.427	47.905	0.6
SiO_2	4.82	TMAO	1000	0	100	0	138.9	131.955	0.6
SiO_2	4.82	TMG*	1	-5.5	1448.13	-1336.6	11.395	10.825	0.6
SiO_2	4.82	TMG*	10	-5.5	9276.36	-7802.63	20.009	19.009	0.6
SiO_2	4.82	TMG*	100	-6	37746.3	-29727.1	50.854	48.311	0.6
SiO_2	4.82	TMG*	1000	-3	4.4E12	-1.3E12	138.59	131.66	0.6
SiO_2	4.82	TMG*	2000	0	100	0	175.39	166.62	0.6

Supplementary Table 30. $U(x)$ **parameter values modelling experiments shown in Fig. 8**

Particle	$2R$ (μm)	Additive	c_b (% or mM)	w (k_BT)	A (k_BT)	B (k_BT)	κ_1 (μm^{-1})	κ_2 (μm^{-1})	A_H (k_BT)
SiO_2	4.82	Tween 20	0.01%	-7	44336.2	-34905.1	11.395	10.825	0.6
SiO_2	4.82	Tween 20	0.1%	-5	30265.2	-23881.3	12.308	11.693	0.6
SiO_2	4.82	Tween 20	0.5%	-5	165586	-120015	18.315	17.399	0.6
SiO_2	4.82	Tween 20	1%	-4	1.94E6	-1.23E6	21.820	20.729	0.6
SiO_2	4.82	Tween 20	2%	-3	6.40E6	-3.76E6	26.926	25.579	0.6
SiO_2	4.82	Tween 20	5%	-1.5	1.49E8	-7.24E7	39.336	37.370	0.6
SiO_2	4.82	control (water)*	0	-6	755482	-512194	38.643	36.710	0.6
SiO_2	4.82	glycerol	0.01	-4.5	7656.6	-6437.35	10.402	9.8821	0.6
SiO_2	4.82	glycerol	0.1	-4.5	9545.7	-7937.66	10.402	9.8821	0.6
SiO_2	4.82	glycerol	1	-4.5	32609.2	-25500.5	10.402	9.8821	0.6
SiO_2	4.82	glycerol	100	-4.5	39283.1	-30434.8	10.402	9.8821	0.6
SiO_2	4.82	glycerol	1000	-4.5	1.03E6	-676592	23.260	22.097	0.6
SiO_2	4.82	glycerol	2000	-2.5	3.61E6	-2164450	31.207	28.086	0.6

* conductivity and p*H* matched to 5% Tween 20 for comparison.

Supplementary references

1 Kubincová, A., Hünenberger, P. H. & Krishnan, M. Interfacial solvation can explain attraction between like-charged objects in aqueous solution. *J. Chem. Phys.* **152**, 104713 (2020).

2 Behjatian, A., Walker-Gibbons, R., Schekochihin, A. A. & Krishnan, M. Nonmonotonic Pair Potentials in the Interaction of Like-Charged Objects in Solution. *Langmuir* **38**, 786−800 (2022).

3 Wang, S., Walker-Gibbons, R., Watkins, B., Flynn, M. & Krishnan, M. A charge-dependent long-ranged force drives tailored assembly of matter in solution. *Nat. Nanotechnol.* **19**, 485–493 (2024).

4 Ninham, B. W. & Parsegian, V. A. Electrostatic potential between surfaces bearing ionizable groups in ionic equilibrium with physiologic saline solution. *J. Theor. Biol.* **31**, 405-428 (1971).

5 Walker-Gibbons, R., Kubincová, A., Hünenberger, P. H. & Krishnan, M. The Role of Surface Chemistry in the Orientational Behavior of Water at an Interface. *J. Phys. Chem. B* **126**, 4697-4710 (2022).

6 Kathmann, S. M., Kuo, I. F. W., Mundy, C. J. & Schenter, G. K. Understanding the Surface Potential of Water. *J. Phys. Chem. B* **115**, 4369-4377 (2011).

7 Remsing, R. C., Baer, M. D., Schenter, G. K., Mundy, C. J. & Weeks, J. D. The Role of Broken Symmetry in Solvation of a Spherical Cavity in Classical and Quantum Water Models. *J. Phys. Chem. Lett.* **5**, 2767-2774 (2014).

8 Hummer, G., Pratt, L. R., García, A. E., Berne, B. J. & Rick, S. W. Electrostatic Potentials and Free Energies of Solvation of Polar and Charged Molecules. *J. Phys. Chem. B* **101**, 3017-3020 (1997).

9 Cox, S. J., Thorpe, D. G., Shaffer, P. R. & Geissler, P. L. Assessing long-range contributions to the charge asymmetry of ion adsorption at the air–water interface. *Chem. Sci.* **11**, 11791-11800 (2020).

10 Grunwald, E. & Berkowitz, B. J. The Measurement and Correlation of Acid Dissociation Constants for Carboxylic Acids in the System Ethanol-Water. Activity Coefficients and Empirical Activity Functions. *J. Am. Chem. Soc.* **73**, 4939-4944 (1951).

11 Belskii, V. E., Kudryavtseva, L. A., Derstuganova, K. A., Teitelbaum, A. B. & Ivanov, B. E. Basicity of Aliphatic-Amines in Ethanol. *Bulletin of the Academy of Sciences of the USSR Division of Chemical Science* **30**, 736-738 (1981).

12 Emami, F. S. *et al.* Force Field and a Surface Model Database for Silica to Simulate Interfacial Properties in Atomic Resolution. *Chem. Mater.* **26**, 2647-2658 (2014).

13 Ide, M. *et al.* Quantification of silanol sites for the most common mesoporous ordered silicas and organosilicas: total versus accessible silanols. *Phys. Chem. Chem. Phys.* **15**, 642-650 (2013).

14 Darlington, A. M. & Gibbs-Davis, J. M. Bimodal or Trimodal? The Influence of Starting pH on Site Identity and Distribution at the Low Salt Aqueous/Silica Interface. *J. Phys. Chem. C* **119**, 16560-16567 (2015).

15 Fan, H.-F., Li, F., Zare, R. N. & Lin, K.-C. Characterization of Two Types of Silanol Groups on Fused-Silica Surfaces Using Evanescent-Wave Cavity Ring-Down Spectroscopy. *Anal. Chem.* **79**, 3654-3661 (2007).

16 Dong, Y., Pappu, S. V. & Xu, Z. Detection of Local Density Distribution of Isolated Silanol Groups on Planar Silica Surfaces Using Nonlinear Optical Molecular Probes. *Anal. Chem.* **70**, 4730-4735 (1998).

17 Lagström, T., Gmür, T. A., Quaroni, L., Goel, A. & Brown, M. A. Surface Vibrational Structure of Colloidal Silica and Its Direct Correlation with Surface Charge Density. *Langmuir* **31**, 3621-3626 (2015).
18 Zhuravlev, L. T. The surface chemistry of amorphous silica. Zhuravlev model. *Colloids Surf., A* **173**, 1-38 (2000).
19 Gaur, A. & Balasubramanian, S. Liquid ethylene glycol: prediction of physical properties, conformer population and interfacial enrichment with a refined non-polarizable force field. *Phys. Chem. Chem. Phys.* **24**, 10985-10992 (2022).
20 Di Mino, C. *et al.* Weak Interactions in Dimethyl Sulfoxide (DMSO)–Tertiary Amide Solutions: The Versatility of DMSO as a Solvent. *J. Phys. Chem. B* **127**, 1357-1366 (2023).
21 Ong, S., Zhao, X. & Eisenthal, K. B. Polarization of water molecules at a charged interface: second harmonic studies of the silica/water interface. *Chem. Phys. Lett.* **191**, 327-335 (1992).
22 Rimola, A., Costa, D., Sodupe, M., Lambert, J.-F. & Ugliengo, P. Silica Surface Features and Their Role in the Adsorption of Biomolecules: Computational Modeling and Experiments. *Chem. Rev.* **113**, 4216-4313 (2013).
23 Bag, S. *et al.* Buffer Influence on the Amino Acid Silica Interaction. *Chemphyschem* **21**, 2347-2356 (2020).
24 Gao, Q. *et al.* Amino Acid Adsorption on Mesoporous Materials: Influence of Types of Amino Acids, Modification of Mesoporous Materials, and Solution Conditions. *J. Phys. Chem. B* **112**, 2261-2267 (2008).
25 Xie, M. & Brüschweiler, R. Degree of N-Methylation of Nucleosides and Metabolites Controls Binding Affinity to Pristine Silica Surfaces. *J. Phys. Chem. Lett.* **11**, 10401-10407 (2020).
26 Mocellin, A., Gomes, A. H. d. A., Araújo, O. C., de Brito, A. N. & Björneholm, O. Surface Propensity of Atmospherically Relevant Amino Acids Studied by XPS. *J. Phys. Chem. B* **121**, 4220-4225 (2017).
27 Herboth, R., Gopakumar, G., Caleman, C. & Wohlert, M. Charge State Dependence of Amino Acid Propensity at Water Surface: Mechanisms Elucidated by Molecular Dynamics Simulations. *J. Phys. Chem. A* **125**, 4705-4714 (2021).
28 Abadian, H. *et al.* Leucine on Silica: A Combined Experimental and Modeling Study of a System Relevant for Origins of Life, and the Role of Water Coadsorption. *Langmuir* **38**, 8038-8053 (2022).
29 Zou, Q., Bennion, B. J., Daggett, V. & Murphy, K. P. The Molecular Mechanism of Stabilization of Proteins by TMAO and Its Ability to Counteract the Effects of Urea. *J. Am. Chem. Soc.* **124**, 1192-1202 (2002).
30 Stasiulewicz, M., Panuszko, A., Bruździak, P. & Stangret, J. Mechanism of Osmolyte Stabilization–Destabilization of Proteins: Experimental Evidence. *J. Phys. Chem. B* **126**, 2990-2999 (2022).
31 Levin, Y. Electrostatic correlations: from plasma to biology. *Rep. Prog. Phys.* **65**, 1577-1632 (2002).
32 Diehl, A. & Levin, Y. Colloidal charge reversal: Dependence on the ionic size and the electrolyte concentration. *J. Chem. Phys.* **129**, 124506 (2008).
33 Winter, E. M. & Groves, J. T. Surface binding affinity measurements from order transitions of lipid membrane-coated colloidal particles. *Anal. Chem.* **78**, 174-180 (2006).
